# Supplementary material for: Synergy between Lactobacillus murinus and anti-PcrV antibody delivered in the airways to boost protection against Pseudomonas aeruginosa
Source: Mol Ther Methods Clin Dev. 2024 Aug 30;32(4):101330. doi: 10.1016/j.omtm.2024.101330 (PMC11418128; doi:10.1016/j.omtm.2024.101330)
Supplement: Document S1. Figures S1–S5 [file mmc1.pdf]

OMTM, Volume *32*

Supplemental information

Synergy between *Lactobacillus murinus* and anti-PcrV antibody delivered in the airways to boost protection against *Pseudomonas aeruginosa*

Thomas Sécher, Mélanie Cortes, Chloé Boisseau, Marie-Thérèse Barba Goudiaby, Aubin Pitiot, Christelle Parent, Muriel Thomas, and Nathalie Heuzé-Vourc'h

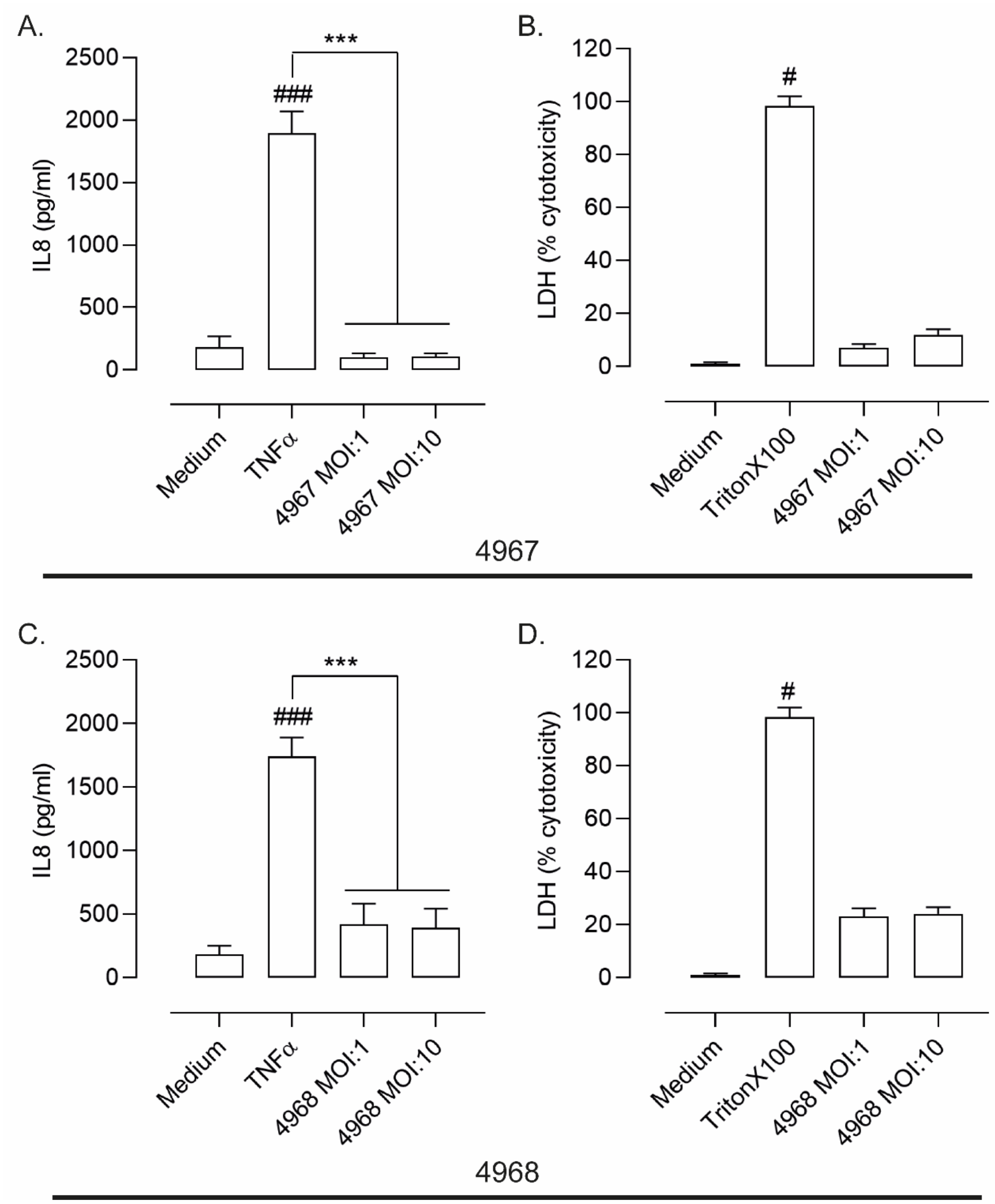

Figure S1: BEAS-2B cells were exposed or not with CNCM-I 4967 (MOI:1 or MOI:10), CNCM-I 4968 (MOI:1 or MOI:10), TNF (1ng/mL) or TritonX100 (10%) for 18h. Production of IL-8 (A and C) and of LDH (B and D) were measured. LDH was expressed as % of response over Triton-X100 conditions (positive control for cell death. The data are quoted as the mean values ± SEM. The results correspond to 3 pooled, independent experiments (n=3-9 per conditions), #: p< 0.05, ###: p<0.001 when compared to medium condition; ***: p<0.001 when compared TritonX100 condition.

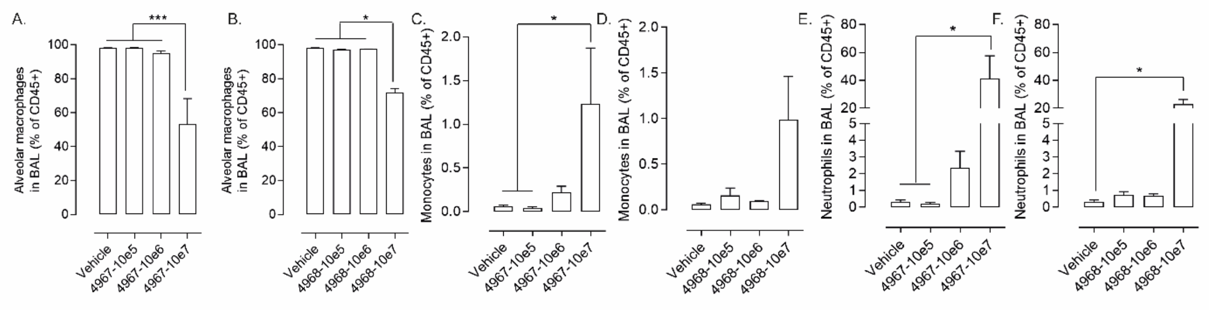

Figure S2: Mice were treated as described in Figure 2A. One day later, mice were sacrificed. Frequency of alveolar macrophages (CD45+ CD11c+ CD11b- SiglecF+ Ly6G- Ly6C- cells) (A and B), monocytes (CD45+ CD11c- CD11b+ SiglecF+ Ly6G- Ly6C+) (C and D) and neutrophils (CD45+ CD11c- CD11b+ SiglecF- Ly6G+ Ly6C-) (E and F) were determined in BAL. The data are quoted as the mean values ± SEM. The results are representative of 2 independent experiments (n=4-5 mice per group), *: $p<0.05$, ***: $p<0.001$ with *t* test.

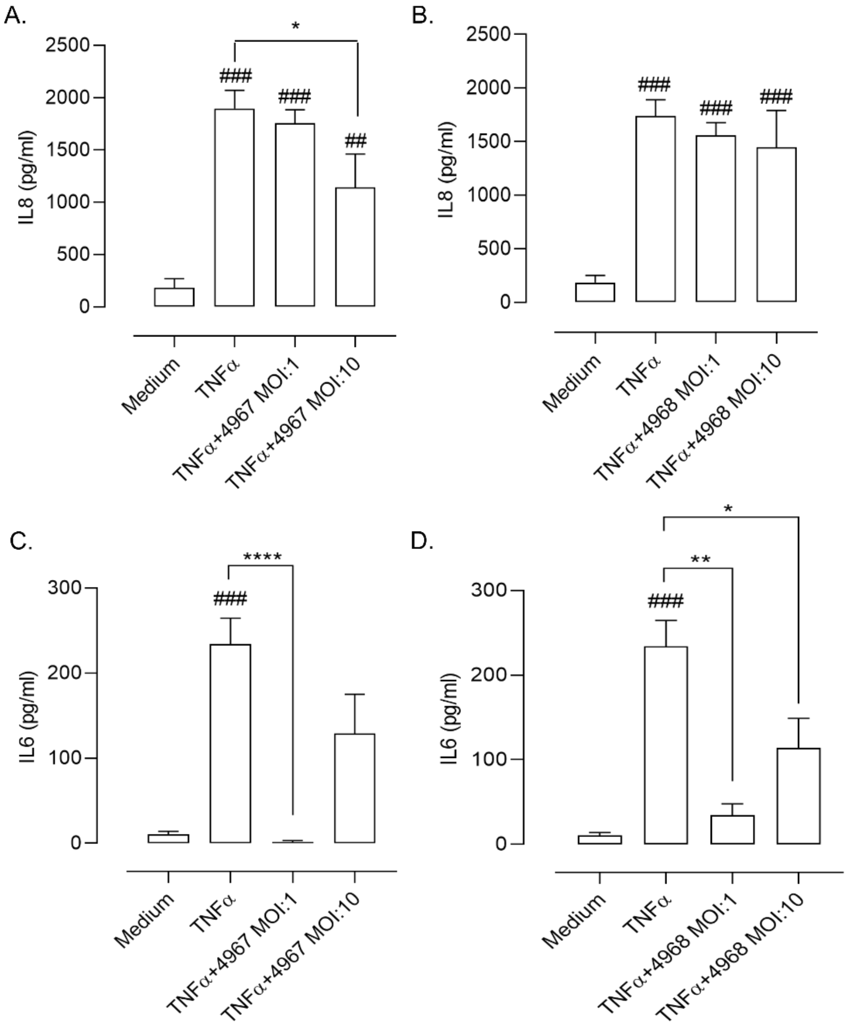

Figure S3: BEAS-2B cells were exposed or not with CNCM-I 4967 (MOI:1 or MOI:10), CNCM-I 4968 (MOI:1 or MOI:10) and/or TNF (1ng/mL) for 18h. Production of IL-8 (A and B) and IL-6 (C and D) was measured. The data are quoted as the mean values ± SEM. The results correspond to 3 pooled, independent experiments (n=3-9 per conditions), ##: $p< 0.01$, ###: $p<0.001$ when compared to medium condition; ***: $p<0.001$ when compared TNF condition.

A.

B.

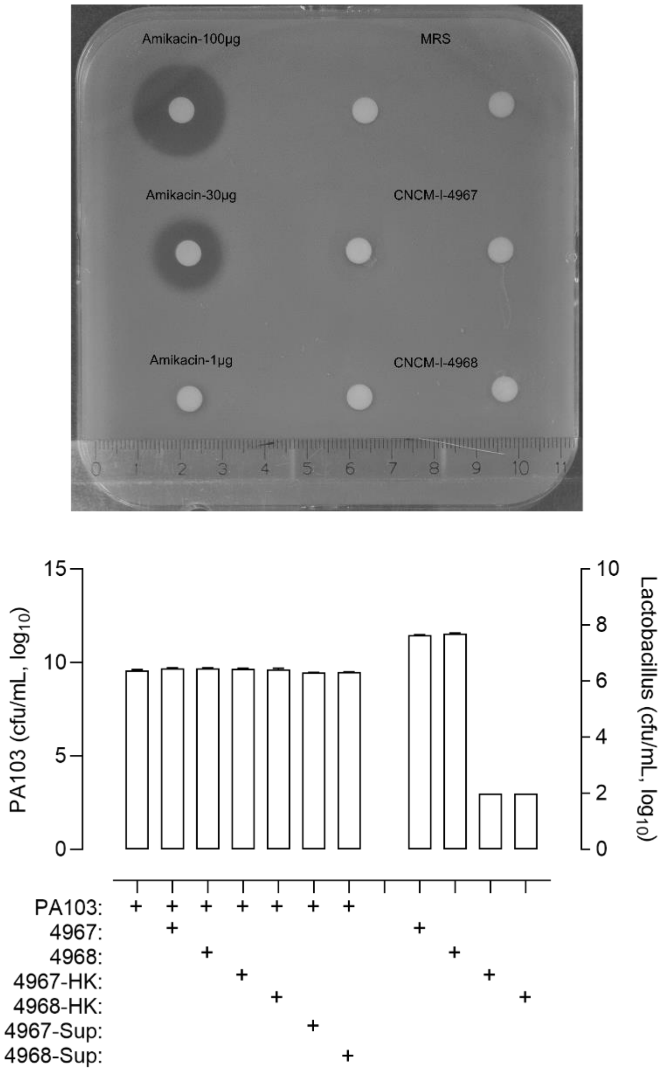

<u>Figure S4:</u> (A) Picture of growth inhibition zone induced by CNCM-I 4967, CNCM-I 4968 or Amikacin (1, 30, 100µg) on *P. aeruginosa* PA103 cultured in soft-agar. (B) CFU counts of *P. aeruginosa* PA103 following a 24-hour co-culture in LB medium with live CNCM-I 4967 (4967), live CNCM-I 4968 (4968), heat-killed CNCM-I 4967 (4967-HK), heat-killed CNCM-I 4968 (4968-HK), supernatant from live CNCM-I 4967 (4967-Sup), supernatant from live CNCM-I 4968 (4968-Sup) have been evaluated on PIA-agar and depicted on the left-side axis. CFU counts of CNCM-I 4967 (4967), live CNCM-I 4968 (4968), heat-killed CNCM-I 4967 (4967-HK), heat-killed CNCM-I 4968 (4968-HK) have been evaluated on MRS-agar and depicted on the right-side axis. The data are quoted as the mean values ± SEM. The results are representative of two independent experiments.

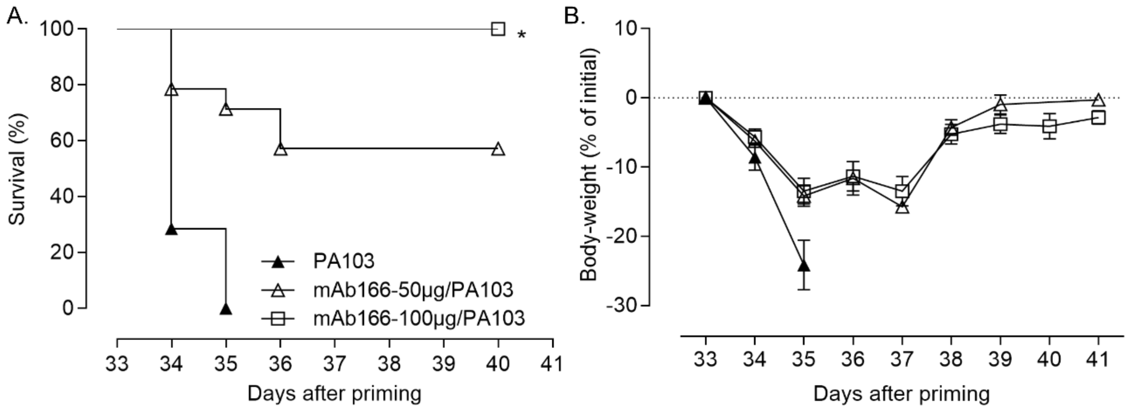

Figure S5: Mice were treated as described in Figure 5A. Survival (A) and body-weight (B) were monitored after the secondary infection. The results correspond to 4 pooled, independent experiments (n=7-20 mice per group). Log-rank test was used for survival analysis, *: p<0.05.
