# Supplementary material for: Synergy between Lactobacillus murinus and anti-PcrV antibody delivered in the airways to boost protection against Pseudomonas aeruginosa
Source: Mol Ther Methods Clin Dev. 2024 Aug 30;32(4):101330. doi: 10.1016/j.omtm.2024.101330 (PMC11418128; doi:10.1016/j.omtm.2024.101330)
Supplement: Document S2. Article plus supplemental information [file mmc2.pdf]

Molecular Therapy
Methods & Clinical Development
Original Article

Synergy between *Lactobacillus murinus* and anti-PcrV antibody delivered in the airways to boost protection against *Pseudomonas aeruginosa*

Thomas Sécher,[1,2] Mélanie Cortes,[1,2] Chloé Boisseau,[1,2] Marie-Thérèse Barba Goudiaby,[3,4] Aubin Pitiot,[1,2] Christelle Parent,[1,2] Muriel Thomas,[3,4] and Nathalie Heuzé-Vourc'h[1,2]

[1]INSERM, Centre d'Etude des Pathologies Respiratoires, U1100 Tours, France; [2]Université de Tours, Centre d'Etude des Pathologies Respiratoires, U1100 Tours, France; [3]Institut Micalis, INRA, AgroParisTech, Université Paris-Saclay, UMR1319 Jouy-en-Josas, France; [4]Paris Center for Microbiome Medicine (PaCeMM), Fédération Hospitalo-Universitaire, Paris, France

Therapeutic antibodies (Ab) have revolutionized the management of multiple illnesses including respiratory tract infections (RTIs). However, anti-infectious Ab displayed several limitations including antigen restrictiveness, narrowed therapeutic windows, and limited dose in the vicinity of the target when delivered by parenteral routes. Strategies enhancing further Ab-dependent containment of infection are currently needed. Here we showed that a combination of inhaled anti-infectious Ab and probiotics is an efficient formulation to protect against lung infection. Using a mouse model of *Pseudomonas aeruginosa*-induced pneumonia, we demonstrated a synergistic effect reducing both bacterial burden and pro-inflammatory response affording protection against primary and secondary infections. This is the first study showing that the local combination in the airways of anti-infective Ab and probiotics subverts suboptimal potency of Ab monotherapy and provides protection against respiratory pathogen.

INTRODUCTION

Respiratory tract infections (RTIs) are one of the leading causes of morbidity and mortality worldwide.[1] RTIs are mostly caused by virus and bacteria and are associated with a significant economic burden. On top of that, the global decline of antibiotic and antiviral effectiveness is recognized as a major threat to human health, by the World Health Organization,[2,3] urging the necessity to develop innovating therapeutic strategies. Therapeutic antibodies (Abs) are used for the treatment of various pathologies including respiratory diseases, with important clinical successes.[4] Moreover, Abs, as a prophylactic or post-exposure treatment, are considered potential game changers for the containment of respiratory infections. Besides Abs, probiotics have emerged as a promising strategy for treating or preventing respiratory tract infections.[5] Probiotics are defined as live bacteria that can confer health benefits to the host when administered in adequate amounts.[6] Probiotics have been proposed to reduce infection rates and the risk of ventilator-associated pneumonia (VAP) in critically ill patients.[7,8] In fact, patients under mechanical ventilation exhibit lung microbiota dysbiosis with increased and low diverse commensal populations precipitating lung infection and pneumonia.[9] Their beneficial effects have been postulated to include enhanced mucosal barrier function, restoration of microbial diversity in established microbiota, modulation of the host inflammatory response and competitive exclusion of pathogens.[10,11] Historically, the lungs have been considered as a sterile organ[12] until recent high-throughput sequencing of bronchoalveolar lavage and pulmonary samples revealed the presence of a core microbiota dominated by *Streptococcus*, *Prevotella*, and *Veillonella* genera. Despite a low biomass, as compared to gut microbiota, airway bacterial communities are involved in critical processes in respiratory health including protection against infections.[13,14] In this context, the therapeutic potential of lung microbiota correctors allowing beneficial microbes to outcompete RTI-associated pathogens may reduce lung inflammation and improve respiratory health.[15,16] However, definitive demonstration of clinical effectiveness of probiotic against respiratory pathogens is still missing while studies also reported negative results[17] requiring optimized treatment strategies.

Abs targeting microbial antigens are usually administered through systemic routes though their beneficial effects are expected in the airways and only a small fraction reaches this compartment after i.v. injection. Over the past decade, we have demonstrated that inhalation constitutes an attractive and feasible alternative route for the delivery of full-length Abs into the lungs.[18–21] Abs delivered through the airways, including mAb166 used in this study, exhibit therapeutic responses and pass poorly and slowly into the systemic circulation.[22] mAb166 is a murine monoclonal IgG2b,κ antibody targeting PcrV, which has been shown to prevent infection in both mice and rat lung infected with *Pseudomonas aeruginosa*.[23,24]

Received 14 December 2023; accepted 27 August 2024;
https://doi.org/10.1016/j.omtm.2024.101330.

Correspondence: Thomas Sécher, INSERM, Centre d'Etude des Pathologies Respiratoires, U1100 Tours, France.
E-mail: thomas.secher@inserm.fr

© 2024 The Author(s). Published by Elsevier Inc. on behalf of The American Society of Gene and Cell Therapy. This is an open access article under the CC BY license (http://creativecommons.org/licenses/by/4.0/).

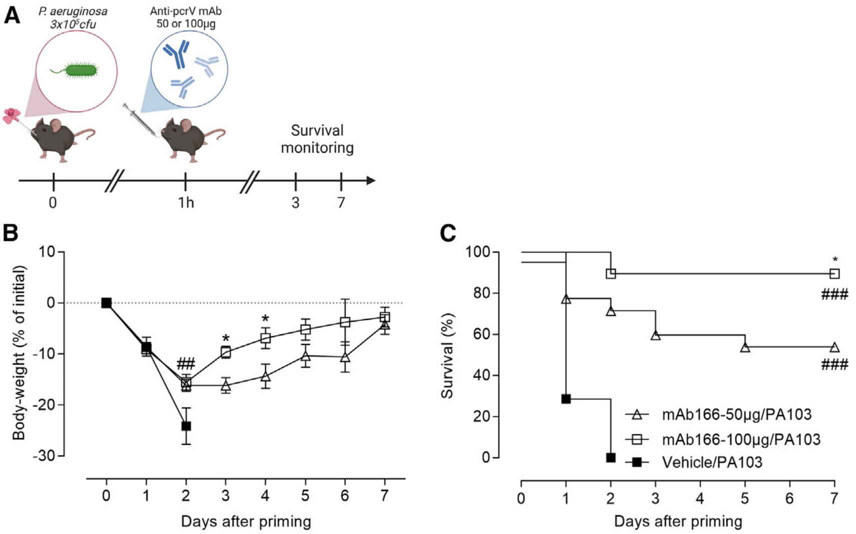

Figure 1. Dose-dependent protection conferred by locally administered anti-*P. aeruginosa* antibody

(A) C57/BL6jRj mice were infected by the orotracheal instillation of 40 µL of *P. aeruginosa* PA103 containing 3×10^5 cfu. One hour later, mice were treated or not with 50 µg or 100 µg of mAb166 via the pulmonary route (airway). Body-weight loss (B) and survival (C) were monitored over 7 days. The results correspond to four pooled, independent experiments (n = 7–20 mice per group). Log rank test was used for survival analysis, ###$p < 0.001$ when compared with Vehicle group; *$p < 0.05$ when compared with mAb166-50-µg group. Two-way ANOVA followed by Tukey's post-test was used for body-weight analysis, ##$p < 0.01$ when compared to Vehicle group; *$p < 0.05$ when compared with the mAb166-50 µg group.

Overall, our findings, supported by other groups, indicate that inhalation may have the edge over other routes of administration if Abs are intended to operate into the respiratory tract to fight respiratory infections.[25–28] Similarly, airway instead of oral application of probiotics would achieve a better therapeutic index while exhibiting fewer systemic effects.[5] In addition, combined use of probiotics with other anti-infectives may improve clinical benefit through mechanistic synergy.

Here, we hypothesized that a combination of anti-infective Ab and probiotics delivered locally into the lungs may better prevent lung infection, than Ab monotherapy. We showed that airway delivery of a neutralizing Ab and *Lactobacillus murinus,* a probiotic isolated from mouse lungs and displaying immunomodulatory properties,[29] provided a synergistic protection against *P. aeruginosa,* a bacterium ranked as a high priority by the World Health Organization.[2] Overall, the combination limited both bacterial burden and pro-inflammatory response. Remarkably, this combination promoted a long-lasting protection against subsequent infections. This is the first study showing that the local combination in the airways of anti-infective Ab and probiotics, subverts suboptimal potency of Ab monotherapy and provides protection against respiratory pathogen.

RESULTS

Dose-dependent protection conferred by airway administered anti-*P. aeruginosa* antibody

We have recently demonstrated that airway administration of mAb166, a murine IgG2b recognizing PcrV and neutralizing type 3 secretion system (T3SS) provided protection from a lethal pulmonary infection with *P. aeruginosa*.[22] Here, we wanted to establish a model of suboptimal mAb166 efficacy. As shown in Figure 1, mice treated with 100 µg of mAb166 were fully protected from this lethal dose while those treated with 50 µg experienced mild symptoms, including significant body-weight loss and evident signs of infection (ruffled fur, hunched posture, and motor impairment), and were partly protected from the infection, as outlined with ∼50% of animal survival (Figures 1B and 1C). Thus, this model (Figure 1A) offers a therapeutic window for the evaluation of combined airway treatment to reinforce Ab efficacy.

Intranasal application of *Lactobacillus murinus* is tolerable in mice

We previously described the immunomodulatory properties of *Lactobacillus* species isolated from lung homogenates of SPF neonatal mice, which mitigated type 2 immunity and the extent of allergic asthma in mice.[29] In the perspective of using probiotics as anti-infectious strategies in combination with Abs, we investigated the effects of daily intranasal applications of two strains of *L. murinus* among our collection of pulmonary bacteria (CNCM-I-4967 and CNCM-I-4968), following the protocol described in Figure 2A. First, we analyzed the extent of probiotics colonization in lungs according to the inoculum size. The two strains displayed a transient, short-term persistence in the airways or in the parenchyma and only for the highest bacterial load (Figures 2B and 2C). Even if strains from the *Lactobacillus* genus are generally regarded as safe (GRAS), we analyzed their impact after intranasal delivery into the lungs. We did not observe an impact on mice body weight (Figures 2D and 2E) nor clinical symptoms (data not shown), whatever the inoculum size. This agrees with the absence of inflammation and cytotoxicity *in vitro* when the probiotics were co-incubated with human bronchial epithelial BEAS-2B cells (Figure S1). However, we observed a significant and dose-dependent recruitment of neutrophils in the airways of mice treated with the strain CNCM-I 4967 and, to a lower and non-significant extent with the strain CNCM-I 4968, 24h after the last administration (Figures 2F and 2G). This was accompanied with an increase of Ly6C + monocytes and concomitant decrease of alveolar macrophage (Figure S2). Overall, our results indicate that intranasally delivered probiotics up to 10^7 colony-forming units (cfu) were well-tolerated by mice. For the rest of the study, the lowest dose (10^5 cfu) of probiotic was selected to avoid confounding immunogenicity during its assessment in combination with anti-infective Abs.

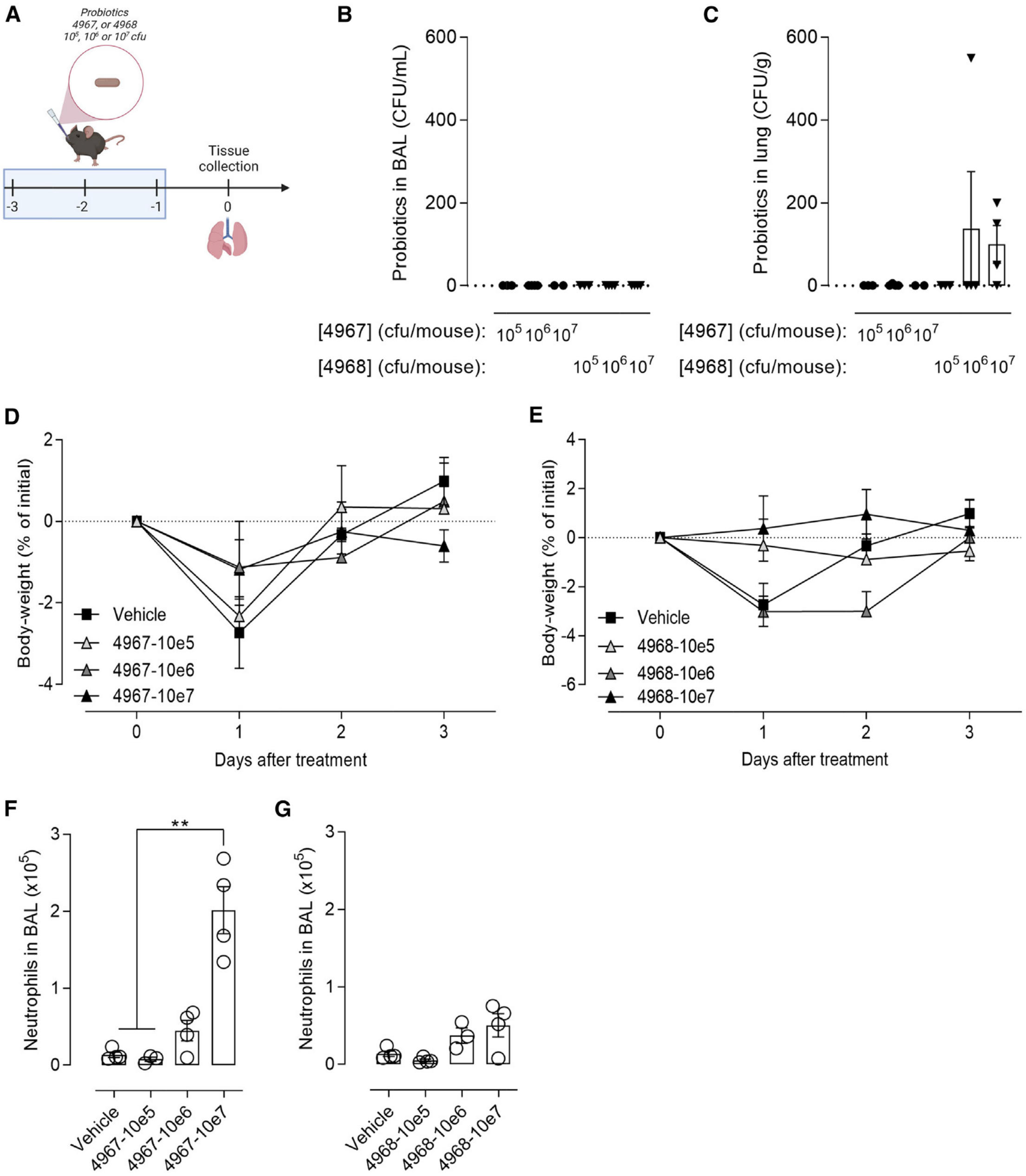

Figure 2. Intranasal application of *Lactobacillus murinus* is tolerable in mice

(A) C57/BL6jRj mice were daily treated or not by the intranasal administration of 40μL of *L. murinus* CNCM-I 4967 or CNCM-I 4968 containing 10^5, 10^6 or 10^7 cfu for 3 consecutive days. One day later, mice were sacrificed. Probiotic load in BAL (B) and lungs (C) were determined. (D and E) Body-weight loss was monitored over 3 days. (F and G) Neutrophils (CD45+ CD11c- CD11b+ SiglecF- Ly6G + Ly6C- cells) number in BAL were evaluated. The data are quoted as the mean values ±SEM. The results are representative of two independent experiments (n = 4–5 mice per group), $^{**}p < 0.01$ with t test.

Synergistic effect of airway-delivered *L. murinus* and anti-infective antibody in *P. aeruginosa*-induced pneumonia

In the absence of adverse effects, combination strategy using two antipseudomonal drugs may be useful against pneumonia.[30,31] Thus, we evaluated the combination of mAb166 and probiotic strains CNCM-I 4967 or CNCM-I 4968 according to the protocol described in Figure 3A. Animals that received either strain CNCM-I 4967 or CNCM-I 4968 were not protected and died within 1–2 days (Figure 3). Notably, mice treated with a suboptimal dose of mAb166 (∼40% of survival) and probiotics survived

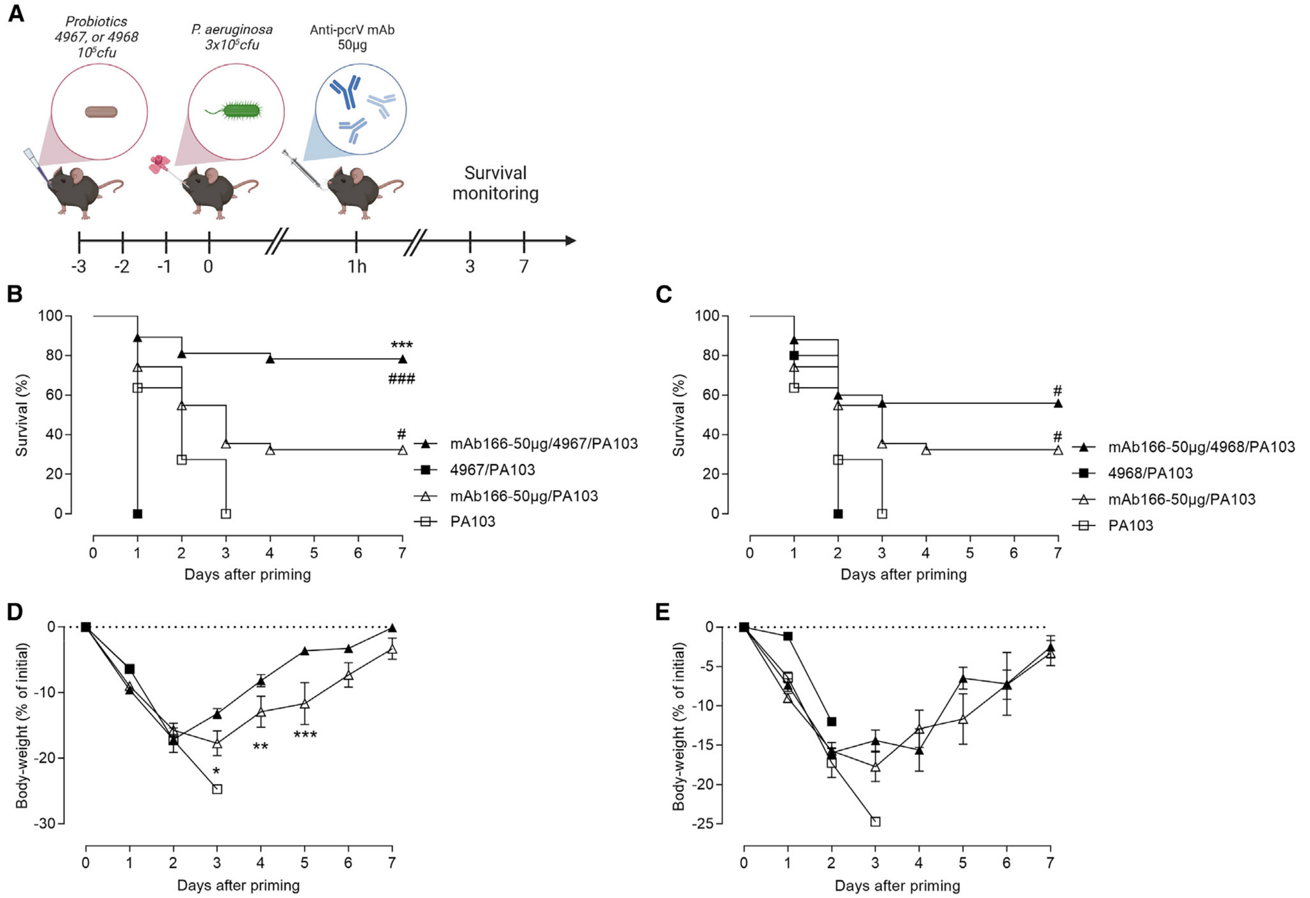

Figure 3. Synergistic effect of intranasal *Lactobacillus murinus* and therapeutic antibody in *P. aeruginosa*-induced pneumonia

(A) C57/BL6jRj mice were daily treated or not by the intranasal administration of 40 µL of *L. murinus* CNCM-I 4967 or CNCM-I 4968 containing 10^5 cfu for 3 consecutive days. One day later, mice were infected and treated as in Figure 1A. Survival (B and C) and body-weight loss (D and E) were monitored over 7 days. The results correspond to four pooled, independent experiments (n = 10–20 mice per group). Log rank test was used for survival analysis, #p < 0.05, ###p < 0.001 when compared to Vehicle group; ***p < 0.01 when compared to mAb166-50µg group. two-way ANOVA followed by Tukey's post-test was used for body-weight analysis, ##p < 0.01 when compared with the Vehicle group; *p < 0.05, **p < 0.01, ***: p < 0.01 when compared to mAb166-50µg group.

the infection better. However, the animals reacted differently to the infection depending on the probiotic strain. Animals that received mAb166 and strain CNCM-I 4967 developed a less severe disease, as illustrated by improved survival and diminished weight loss (Figures 3B and 3D), while mice treated with mAb166 and strain CNCM-I 4968 had similar disease progression and severity as mAb166-only treated animals (Figures 3C and 3E). This suggests different intrinsic properties of the two probiotics.

Improved host anti-bacterial response afforded by airway-delivered *L. murinus* and anti-infective antibody

To determine the mechanisms underlying the protection associated with the combined treatments, we analyzed the lung bacterial load and local inflammatory response. All groups that received mAb166 (alone or in combination) had a significant reduction of *P. aeruginosa* PA103 load in the lungs, as anticipated (Figures 4A, 4B, 4F, and 4G). Based on agar-well diffusion assay and competition assay, we can rule out that the control of *P. aeruginosa* burden is attributable to a direct effect of probiotics on *P. aeruginosa* PA103 as CNCM-I 4967 and -I 4968 did not exhibit a signification ability to inhibit *P. aeruginosa* PA103 growth *in vitro* as compared with control antibiotic or medium, even if the probiotics were live, heat-killed or considering soluble-derived factors (Figure S4). Next, we analyzed the recruitment of neutrophils, which are the main leukocyte subtypes engaged in the lungs to control invading pathogens, in the airways and lung parenchyma.[32,33] After 24 h of infection, there were significantly more neutrophils in both bronchoalveolar lavage (BAL) and lungs of animals that received mAb166 and strain CNCM-I 4967, as compared with mAb166 alone and controls (Figures 4C and 4D). Neutrophilic-dependent anti-bacterial response is necessary for controlling *P. aeruginosa* infection; however, a protracting and non-resolving inflammatory response may be deleterious in experimental lung infection models.[34] Interleukin (IL)-6, a prototypal pro-inflammatory cytokine, was

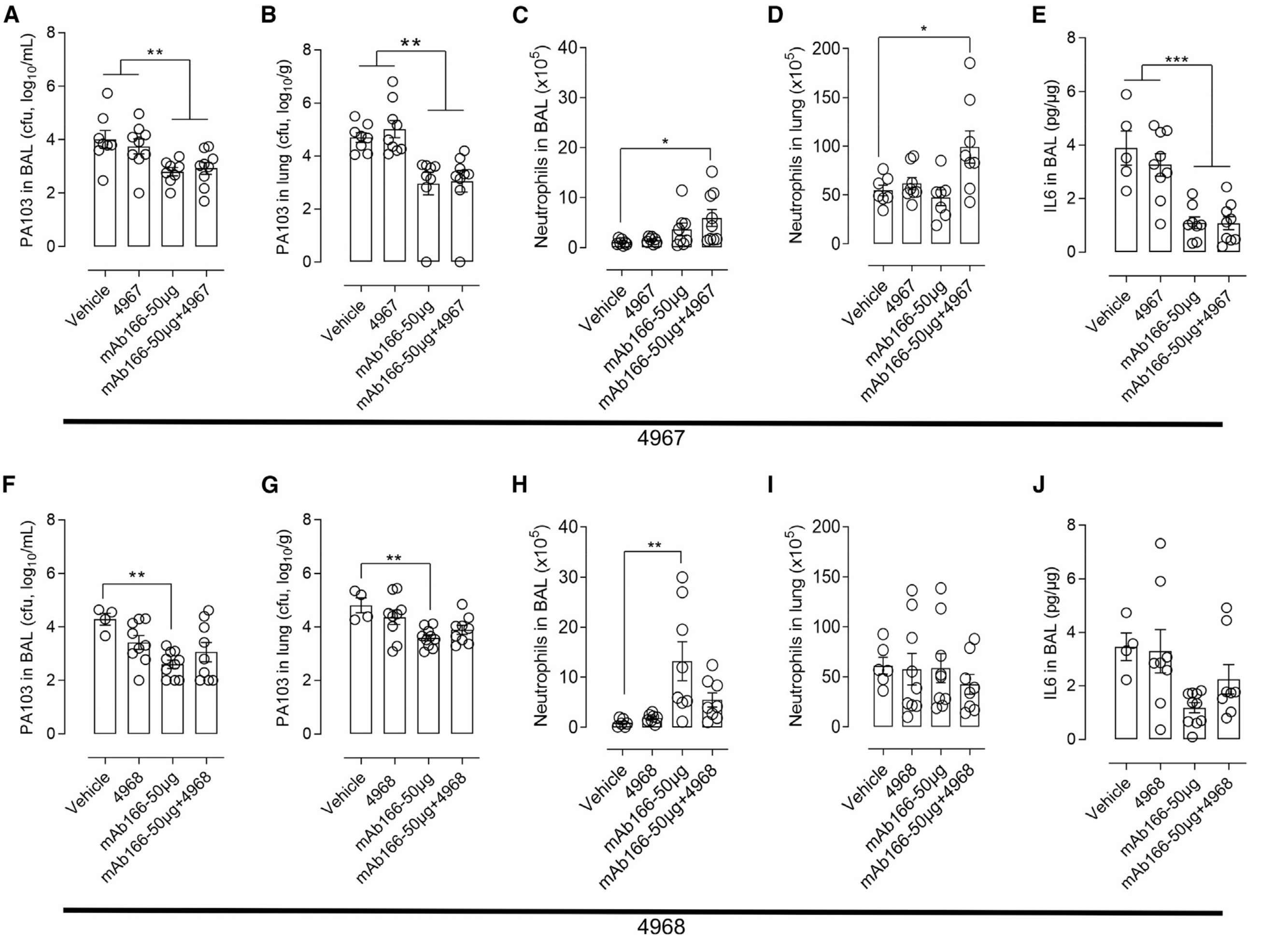

Figure 4. Improved host anti-bacterial response afforded by intranasal Lactobacillus murinus and therapeutic antibody
C57/BL6jRj mice were treated and infected as described in Figure 3A. One day later, mice were sacrificed. *P. aeruginosa* PA103 burden in BAL (A and F) and in lungs (B and G) were quantified. Neutrophils (CD45+ CD11b+ SiglecF- Ly6G + cells) number in BAL (C and H) and in lungs (D and I) were determined. IL6 in BAL (E and J) was measured and standardized over total BAL protein. The data are quoted as the mean values ±SEM. The results correspond to two pooled, independent experiments (n = 10 mice per group), *: $p < 0.05$, **: $p < 0.01$, ***: $p < 0.001$ with one-way ANOVA followed by Tukey's post-test.

significantly reduced in the BAL of the CNCM-I 4967-treated groups as compared with controls (Figure 4E). The better control of inflammation in mAb166 and CNCM-I 4967 strain-treated groups may partially account from probiotic treatment, as CNCM-I 4967 significantly and dose-dependently reduced tumor necrosis factor (TNF)-induced inflammation in BEAS-B cells (Figure S3A), which is not the case for CNCM-I 4968 (Figure S3B). Interestingly, neither neutrophil recruitment nor local inflammation were significantly impacted by strain CNCM-I 4968 when combined with mAb166 (Figures 4F–4J and S3B). Altogether, our results suggest that the combination of probiotics and anti-infective Ab administered locally in the airway improved host anti-*P. aeruginosa* immune responses promoting subsequent infection control after primary exposure. This effect was specific to the probiotic strain. The molecular determinants accounting for this protection remain to be addressed.

The combination of airway-delivered *L. murinus* and anti-infective antibody also promoted long-term protection against *P. aeruginosa*

We have recently demonstrated that airway administration of the anti-*P. aeruginosa* antibody mAb166 promoted genuine innate and adaptive immune responses conferring protection from a secondary bacterial infection.[35] The long-term protection was dependent on the amount of mAb166 with 100 µg providing 100% animal survival after the secondary infection while 50 go was suboptimal with 50% of survival (Figure S5). Here, we investigated the combination of probiotics with suboptimal dose of mAb166 on the protection to secondary infection, according to the protocol described in Figure 5A. All mice treated with the combination of CNCM-I 4967 and mAb166 survived after a secondary infection by *P. aeruginosa*, while one mouse over five in the CNCM-I 4968-mAb166 group died (Figures 5B and 5C). This modest but significant improvement of survival was associated with

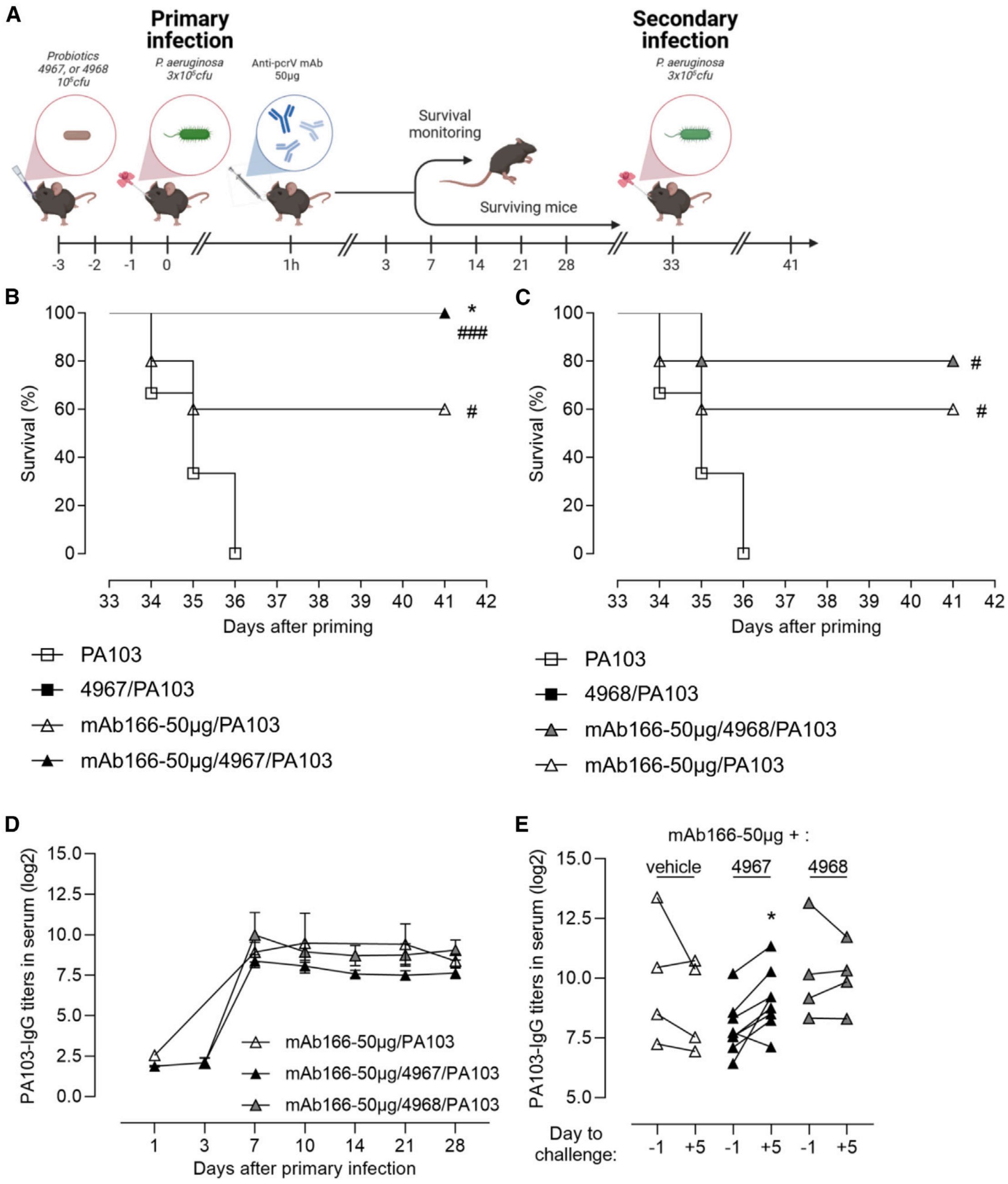

Figure 5. Combined intranasal *Lactobacillus murinus* and therapeutic antibody promotes long-term protection against *P. aeruginosa*

(A) C57/BL6jRj mice were treated and infected as described in Figure 3A (primary infection). Surviving mice were challenged, 33 days later (secondary infection) by an orotracheal instillation of 40 μL of *P. aeruginosa* PA103 (3×10^5 cfu) without additional treatment. (B and C) Survival was monitored over a week after the challenge. The results correspond to four pooled, independent experiments (*n* = 10 for CNCM-I-4967 group and *n* = 5 for the others). Log rank test was used for survival analysis, #: $p < 0.05$, ###: $p < 0.001$ when compared to Vehicle group; *: $p < 0.05$ when compared to mAb166-50μg group. (D) The concentration of total anti- *P. aeruginosa* PA103 IgG in serum was determined by ELISA at days 1, 3, 7, 14, 21, and 28 after the primary infection. The data are quoted as individual values. The results correspond to two pooled experiments (n = 4–15 mice per group). (E) The concentration of total anti-*P. aeruginosa* PA103 IgG in serum was determined by ELISA at days −1, and +5 after the secondary infection. The data are quoted as individual values. The results correspond to two pooled experiments (*n* = 4 for mAb166-50 μg and CNCM-I 4968-mAb166-50μg group and *n* = 8 mice for CNCM-I 4967-mAb166-50μg group), *: $p < 0.05$; with a paired t test.

induction of a memory anti-*P. aeruginosa* humoral response, an immunological hallmark of long-term protection,[35] with mice treated with CNCM-I 4967 and mAb166 producing more anti-*P. aeruginosa* PA103 IgG after the challenge, but not the priming, as compared with the other groups (Figures 5D and 5E). Even if these results need to be confirmed in a bigger cohort, they confirmed previously published work using optimal mAb166 monotherapy.[35]

DISCUSSION

Despite considerable investments in research and development over recent years, only a few anti-infective Abs have been approved so far. Several explanations may account for Ab's suboptimal capacity to treat respiratory pathogens. First, most anti-infective antibodies are monovalent, binding to one antigen, which may be insufficient to neutralize pathogens expressing a vast array of virulence factors or regulating the expression of their antigens during infection. Second, the therapeutic window is critical for the efficacy of Ab immunotherapy, Abs work best when they are administered immediately after diagnosis. Finally, the dose of Ab reaching the infected tissue is paramount for effectiveness. Direct delivery of anti-infective Abs into the lungs, by inhalation, is a promising alternative to conventional intravenous route of administration of Abs for the treatment of pulmonary diseases. Our group and others have demonstrated enhanced airway concentration, increased onset of action, and limited systemic exposure associated with Ab delivery into the lungs.[18–21] Moreover, airway-delivered Abs were associated with better therapeutic response in various models of respiratory infections.[25,27,28,36] Despite these theoretical advantages of local administration for Ab by inhalation, few of these benefits have materialized in the clinics.

Evidence in the literature suggests that combining Ab treatments with other therapies might strengthen pathogen containment, as illustrated for Abs and antibiotics against respiratory pathogens.[31,37,38] Here, we tested the combination of anti-infective Ab with probiotics from the genus *Lactobacillus*, as they have shown to harness lung responses to promote sterilizing immunity as well as inducing long-term adaptive responses.[39–41] Based on the literature showing the benefits of local delivery of Ab to treat respiratory infections,[42–44] both the Ab and the probiotics were delivered locally into the lungs. To our knowledge, our study is the first to examine the effects of intranasal administration of probiotics in combination with inhaled therapeutic antibody against *P. aeruginosa* infection.

In this study, probiotics (CNCM-I 4967 and 4968) were commensals isolated from a neonatal mouse lung.[29] While probiotics and especially *Lactobacillus* have proven safety when administered by the oral route,[45,46] only scarce data are available after application in the respiratory tract. This is of particular importance when considering the usual large dose of probiotic ($\sim 10^8$cfu), necessary to overcome colonization resistance,[47] with the total biomass of the lung microbiota, estimated to be around 10^3–10^5 cfu. When given intranasally, we showed that *Lactobacillus* dose-dependently induced airway inflammation exemplified by the increased recruitment of neutrophils and the concomitant disappearance of alveolar macrophage. For the highest dose, we observed a very limited amount of probiotic remaining in the respiratory tract 24 h after the last administration, suggesting the absence of durable lung colonization. Even if preclinical[29] and clinical data[48] showed an association between lactobacilli colonization of the airways and protective effect against asthma, it remains uncertain whether the intranasal administration of *Lactobacillus* may interfere with lung microbial homeostasis, which could induce severe outcomes.[49,50] For these reasons, we selected a mild quantity of probiotics, to be tested in combination with the Ab, that do not affect body weight nor induce any visible symptoms, assuming tolerability both *in vivo* and *in vitro* and that do not provide protection per se against pulmonary infection.

It is now well established that commensal microbes can control the maturation/functionality of immune cells and modulate the outcome of the immune response.[51] Despite numerous data supporting the use of probiotics in treating gastrointestinal diseases, much less is known about their impact in respiratory diseases. Here, we observed a better clearance of lung *P. aeruginosa* 24 h after infection associated with an enhanced recruitment of neutrophils and a diminished pro-inflammatory response when animals were treated with CNCM-I 4967 and mAb166. Massive lung neutrophil recruitment is a shared downstream mechanism of a protective anti-*P. aeruginosa* immunity and is even more important than the contribution of other immune cell types.[33,52] Indeed, a favorable outcome of *P. aeruginosa,* and more generally lung bacterial, infection necessitates a controlled neutrophil recruitment that will clear the pathogen and promote survival without excessive inflammation. In fact, during pneumonia, a protracted inflammation can lead to exacerbated tissue injury that is ultimately deleterious for the host.[53] Our *in vivo* and *in vitro* results indicate that improved control of *P. aeruginosa* load by combined administration in the airways of probiotic and Ab results in reduced pathogen-mediated inflammation associated with improved survival. Exclusion of pathogens by probiotics is known to be mediated by their ability to compete for nutrients, produce bacteriocin, or modulate the microenvironment.[54,55] In our study, we did not observe any direct bactericidal effect of CNCM-I 4967 over *P. aeruginosa* nor any competitive effect suggesting that its positive effect may rely on immunomodulation, as shown for other *Lactobacillus* strains.[13]

We have recently demonstrated that the local delivery of Abs into the lungs was associated with the promotion of long-term protection against *P. aeruginosa*.[35] However, the vaccine-like effect mediated by mAb166 was dependent on the dose of the bacteria and Ab, which is not satisfactory to translate into the clinic. The analysis of the immune response suggested that the intensity/specificity of the immune response was critical for an optimal Ab-mediated long-term protection against *P. aeruginosa*.[35] Here, we showed that intranasal administration of probiotics compensates the efficacy of suboptimal concentration of mAb166 providing a better long-term protection against RTI-associated pathogens than Ab monotherapy. As previously observed,[35] this may be associated with the induction of a memory adaptive humoral response against *P. aeruginosa,* which composition and specific protective potency remains to be determined. Increasing

preclinical and clinical evidence indicates that the microbiota modulates responses to Abs through interactions with the host immune system.[56,57] Accordingly, we hypothesize that *Lactobacillus* probiotics might potentiate the immune response through the improvement of neutrophil recruitment and the control of the inflammatory response and act synergistically with the neutralizing anti-infective Ab. The fact that intranasal *Lactobacillus* had immunomodulatory functions in lung infection models further supports this hypothesis.[13,58,59] Several studies have suggested that probiotics may activate specific T cell responses against microbial antigens that either potentiate pathogen-specific immune activation or may even induce heterologous protective responses.[60–63]

The prevention or treatment of respiratory tract infections has been complicated after decades of excessive use and misuse of broad-spectrum antibiotics and the epidemic increase in antibiotic resistance. This has significantly reduced the effectiveness of treatments, altering patient outcomes and placing considerable pressure on overburdened health care systems. The prevalence of *P. aeruginosa* infections has increased over the past decade and has become a major concern in the hospital environment. Indeed, *P. aeruginosa* is one of the most common etiologic agents of intensive care unit infections and is among the leading causes of VAP due to mechanical ventilation, trauma, or antecedent viral infections with a significantly higher attributable mortality as compared with other pathogens.[64,65] *P. aeruginosa* is also intrinsically associated with chronic pulmonary diseases and antibiotic resistance.[66] Finally, resolving pneumonia requires not only to fight against airway contamination by exogenous pathogen, but also against ecological dysbiosis[9] that fuels long-lived lung dysfunctions.[67] In this context, the development of innovative targeted host-directed immunotherapy holds great promise. Additional experiments will be necessary to provide mechanistic insights on the synergistic antibacterial effects of probiotic and therapeutic Ab administered locally into the airways after both primary and secondary infections, and to extend these results to other infectious agents and Abs. However, this strategy may offer novel preventive or therapeutic opportunities against pneumonia.

MATERIALS AND METHODS

Mice

Adult male C57BL/6jrj (B6) mice (6–8 weeks old) were obtained from Janvier (Le Genest Saint-Isle, France). All mice were housed under specific-pathogen-free conditions at the PST “Animaleries” animal facility (Université de Tours, France) and had access to food and water *ad libitum*. All experiments complied with the French government's ethical and animal experiment regulations (APAFIS#7608–2016111515206630).

P. aeruginosa infection

PA103 (ATCC 33348) *Pseudomonas aeruginosa* strain, kindly provided by Pr. Teiji Sawa (Kyoto University, Japan) was used in this study. The uniformity of the colonies was checked by plating on *Pseudomonas* isolation agar (PIA) plates. PA103 has been transformed by quadriparental mating by a mini-Tn7T transposon encoding allowing a constitutive expression of the *LuxCDABE* operon. Bacteria were prepared as previously described.[27] Mice were anesthetized with isoflurane 4% and an operating otoscope fit with intubation specula was introduced to both maintain tongue retraction and visualize the glottis. A fiberoptic wire threaded through a 20G catheter and connected to the torch stylet (Harvard Apparatus, France) was inserted into the mouse trachea. Correct intubation was confirmed using lung inflation bulb test and 40 μL of the bacterial solution at 3.10^5 cfu was applied using an ultrafine pipette tip. Inoculum size for infections were confirmed by counting colony-forming units (cfu) on PIA plates. Mortality and body weight of animals were monitored daily. In all experiments, moribund animals, or animals with a weight loss of more than 20% were euthanized for ethical reasons and considered as dead animals due to the infection. For long-term experiments, an additional control group of mice that were solely infected with *P. aeruginosa* PA103 was added to confirm the lethality of the inoculum.

Antibody administration

mAb166 was generated from PTA-9180 hybridoma (LGC Standards, France) and supplied as sterile, non-pyrogenic, PBS solution under good manufacturing practice (BioXcell, USA). mAb166 was administered 1 h after the infection, at the dose of 100 μg or 50 μg (suboptimal quantity) in 50 μL of PBS using a Microsprayer aerosolizer (Penn-Century, USA) introduced orotracheally, as described in the previous section.

Probiotic administration

Probiotics strains used in this study were previously isolated from neonatal mouse lung homogenate.[29] They were deposited at the French National Collection of Microorganism Cultures (CNCM) under the name CNCM-I-4967 and CNCM-I-4968 (patented strains). Based on its genomic sequence (Illumina sequencing technology provided by Eurofins), both strains were identified as *Lactobacillus murinus* or *Ligilactobacillus murinus* according to the most recent taxonomy.[68] The genome analysis was performed using the PathoSystems Resource Integration Center (PATRIC, Version 3.5.31, https://www.bv-brc.org/) and Rapid Annotation using Subsystem Technology (RAST, Version 2.0, https://rast.nmpdr.org/). In PATRIC, the services Assembly, Annotation, Comprehensive Genome Analysis, and BLAST were used. In RAST, the tools Subsystem Feature Count, Function-Based Comparison, and Sequence-Based Comparison were used. Starting from frozen stock solution at −80°C, strains were resuscitated on Man, Rogosa, Sharpe (MRS)-agar plate and put in culture overnight in 13 mL of liquid MRS in a 14-mL Falcon tube at 37°C, 5% CO_2 without shaking. Bacteria were washed once in PBS diluted in saline, to obtain a titer of 10^5 bacteria/40 μL. Mice were anesthetized with isoflurane 4% and 40 μL of the bacterial solution or the corresponding vehicle solution was applied intranasally using an ultrafine pipette tip. Mice were left to recover for 10 s in an upright position before being put back in their cage. Each inoculum was then checked for accuracy and uniformity (by direct plating on fresh MRS-plates).

BAL, organ sampling, bacterial load assay

Bronchoalveolar lavage fluid (BAL) was collected after *P. aeruginosa* infection by introducing a catheter into the trachea under deep pentobarbital anesthesia and washing sequentially the lung with 1 × 0.5 mL and 2 × 1 mL of 1X PBS at room temperature. The lavage fluid was centrifuged at 400 × g for 10 min at 4°C and the supernatant of the first lavage was stored at −20°C for analysis. The cell pellet was resuspended in PBS, counted in a hemocytometer chamber, and used for subsequent analysis. Lungs were perfused with 10 mL of PBS1X and harvested in GentleMACS C tubes (Miltenyi Biotec, Germany) containing 2 mL of RPMI medium (Invitrogen, France) for flow cytometry or GentleMACS M tubes (Miltenyi Biotec, Germany) containing 2 mL of 1X PBS for microbiology assay. Bacterial load in lung homogenates or BAL (before centrifugation) was determined by plating 10-fold serial dilutions on PIA-agar or MRS-agar. Plates were incubated at 37°C in a 5% CO_2 atmosphere, and the cfu were counted after 24 h.

Preparation of pulmonary immune cells

Lung and spleen homogenates were prepared using a GentleMACS tissue homogenizer (Miltenyi Biotec, Germany). Lung pieces were then digested in a medium containing 125 μg/mL of Liberase (Roche, France) and 100 μg/mL of DNAse I (Sigma, France) for 30 min at 37°C under gentle agitation. After washes, contaminating erythrocytes were lysed using Hybri-Max lysis buffer (Sigma, France) according to the manufacturer's instructions. Samples were sequentially filtered over 100-μm and 40-μm nylon mesh. After final wash, cell pellets were resuspended in PBS containing 2% FCS, 2 mM EDTA, and 1X murine Fc-block (Becton Dickinson, France)—described elsewhere as FACS buffer.

Flow cytometry

Cells were incubated in FACS buffer for 20 min at 4°C. Then, cells were stained in FACS buffer for 20 min at 4°C with appropriate dilutions of following antibodies: CD45-APC-Cy7 (30-F11), CD11b APC (M1/70), Ly6G FITC (1A8), CD11c PE (N418), Ly6C Pe-Cy7 (HK1.4) from Biolegend and Siglec-F BV421 (E50-2440) from BD. Dead cells were stained with the LIVE/DEAD Fixable Aqua Dead Cell Staining kit (Thermo Fisher Scientific) and acquired on an MACS Quant (Miltenyi Biotec) cytometer. Analyses were performed using Venturi-One software (Applied Cytometry; UK). Alveolar macrophages were defined as follows: CD45+ CD11c+ CD11b- SiglecF+ Ly6G− Ly6C−, neutrophils were defined as follows: CD45+ CD11c− CD11b+ SiglecF− Ly6G+ Ly6C−, and monocytes were defined as CD45+ CD11c− CD11b+ SiglecF + Ly6G− Ly6C+.

BEAS-2B human bronchial epithelial cell challenge

BEAS-2B human bronchial epithelial cell line was obtained from the American Type Culture Collection (ATCC CLR-9609). The protocol of co-incubation was adapted from previous publication.[14] Cells were maintained in RPMI-1640 medium supplemented with 10% fetal bovine serum (Eurobio, Les Ulis, France), 1% penicillin/streptomycin (Sigma-Aldrich, St. Louis, MO, USA) and 1% L-glutamine (Gibco, Thermo Fisher Scientific, Waltham, MA, USA) in 75 cm^2 tissue culture flask (Sarstedt, Nümbrecht, Germany) and incubated at 37°C, in a 5% CO_2 atmosphere, in a humidified incubator. Cells were passaged before reaching 80% confluency, using Trypsin-EDTA (Gibco, Thermo Fisher Scientific, Waltham, MA, USA). Cells were treated, as indicated with 1 ng/mL TNF-α, and/or living 4967 and 4968 bacteria (MOI 1:1 and 1:10) in RPMI-1640 medium supplemented with 10% fetal bovine serum and 1% L-glutamine. After 6 h of co-incubation, supernatants were collected to measure cytokines and lactate dehydrogenase (LDH) release (cell viability control). Production of IL-8 in BEAS-2B culture supernatants was examined using a commercially human IL-8 ELISA kit (Fisher Scientific) according to the manufacturer's recommendation. The Colorimetric assay that quantitatively measures lactate dehydrogenase (LDH) released upon cell lysis was CytoTox 96 (Promega).

Agar-well diffusion method

The agar-spot method was adapted from our previous studies.[14,69,70] Briefly, the capacity of the two CNCM-I 4967 and CNCM-I 4968 strains to inhibit the growth of *P. aeruginosa* PA103 was evaluated by measuring the diameter of inhibition of the culture relative to that of antibiotic (amikacin sulfate, 30μg/well). A layer of 40 mL MRS agar was poured into a 12-cm-square Petri dish and allowed to cool down, then an equal volume (20 μL) of each CNCM-I 4967 and CNCM-I 4968 overnight bacterial suspension was deposited on cellulose disks. A second layer of 20 mL lysogeny broth (LB) agar containing *P. aeruginosa* PA103 or PA14 (McFarland = 1) culture was added on top. The plates were incubated at 37°C and the growth inhibition zones were measured 24 h later. The inhibition diameters obtained with 4967 and 4968 smaller to that of the reference antibiotic control were considered to have a negative antagonistic effect.

Competitive growth assay

PA103 was grown in lysogeny broth (LB Lennox, Invitrogen) overnight at 37°C with shaking at 200 rpm. CNCM-I 4967 and CNCM-I 4968 were grown in 13 mL of liquid MRS overnight in a 14-mL Falcon tube at 37°C without shaking. The following preparations were used to evaluate PA103 inhibition by probiotics: (1) whole-cell culture containing 106 cfu/mL of CNCM-I-4967 or 4968, (2) whole-cell culture containing 106 cfu/mL of CNCM-I-4967 or 4968 after being heat-killed (30 min at 37°C), (3) culture supernatants from whole-cell culture containing 106 cfu/mL recovered following centrifugation and filtration through 0.22-μm filters and buffered to pH 7. One milliliter of both competing (CNCM-I-4967 or CNCM-I-4968 from different preparations), and target strains (PA103) were inoculated together for 24 h at 37°C without shaking as previously described.[71–73] PA103 CFU were enumerated, by serial-dilution in PBS and plated on selective PIA agar plates for 24 h at 37°C. As controls, the growth of competing strains was systematically checked on MRS-agar plates. The following conditions were tested: PA103 alone, PA103 co-cultured with live CNCM-I-4967 or CNCM-I-4968, PA103 co-cultured with heat-killed CNCM-I-4967 or CNCM-I-4968 (30 min at 70°C), PA103 co-cultured with buffered supernatant (pH = 7) from CNCM-I-4967 or CNCM-I-4968.

Cytokine measurements and IgG measurements

P. aeruginosa IgG titers, in serum and BALF levels were measured by sandwich ELISA. Briefly, high-binding Immulon 96-well plates (ThermoFischer Scientific, France) were coated with 0.5 μg/mL of *P. aeruginosa* PA103 lysate, prepared from overnight cultures that were then sonicated, and diluted in bicarbonate buffer. The plates were then washed and blocked with 1%BSA-PBS. Serum or BAL samples were incubated for 2 h. A biotin-conjugated goat anti-rat IgG antibody (Biolegend, France) was added for 2 h. After a washing step, peroxidase-conjugated streptavidin (R&D) was added for 20min. Between each step, plates were thoroughly washed in 0.05%-Tween20-PBS. Tetramethylbenzidine was used as a substrate, and the absorbance was measured at 450 nm using a microplate reader (Tecan, Switzerland). The titer was calculated by binary logarithm regression as the reciprocal dilution of the sample, where the extinction was 2-fold the background extinction. Cytokine concentrations in BAL were assessed using a sandwich ELISA according to manufacturer's instructions (Biotechne, France) and standardized over total BAL protein.

Statistical analysis

Statistical evaluation of differences between the experimental groups was determined by using one-way analysis of variance (ANOVA) followed by a Newman-Keuls post-test (which allows comparison of all pairs of groups). Log rank test was used for survival analysis. Student's t test was used for comparison between two groups and paired t test was used when comparing the same individual before and after challenge. All tests were performed with GraphPad Prism, Version 8 for Windows (GraphPad Software Inc., San Diego, CA, USA; www.graphpad.com). All data are presented as mean +/− standard error of the mean (SEM). A p-value < 0.05 was considered significant.

DATA AND CODE AVAILABILITY

The data generated in this study are available upon request from the corresponding author.

ACKNOWLEDGMENTS

This work was supported by a public grant overseen by the French National Research Agency (ANR) as part of the "Investissements d'Avenir" program (LabEx MabImprove, ANR-10-LABX-53-01). Additional fundings were provided by Region Centre-Val-de-Loire (Novantinh Program) and C-VALO (Infinhitim Program).

AUTHOR CONTRIBUTIONS

T.S. and N.H.-V. conceived the study. All authors substantially contributed to the acquisition, analysis, or interpretation of data. M.T. provided probiotic strains and expertise in the analysis of their immunomodulatory functions. T.S., M.T., and N.H.-V. contributed to manuscript drafting, revising and, critically reviewing. All authors approved the final version of this manuscript to be published.

DECLARATION OF INTERESTS

N.H.-V. is co-founder and scientific expert for Cynbiose Respiratory. In the past 2 years, she received consultancy fees from Eli Lilly, Argenx, and Novartis and research support from Sanofi and Aerogen Ltd. M.T. is co-founder of the start-up Carembouche.

SUPPLEMENTAL INFORMATION

Supplemental information can be found online at https://doi.org/10.1016/j.omtm.2024.101330.

REFERENCES

1. European Respiratory Society (2016). The burden of lung disease (European Respiratory Society).
2. World Health Organization (2017). WHO Publishes List of Bacteria for Which New Antibiotics Are Urgently Needed at (World Health Organization).
3. World Health Organization (2020). The top 10 causes of death (World Health Organization).
4. Mayor, A., Chesnay, A., Desoubeaux, G., Ternant, D., Heuzé-Vourc'h, N., and Sécher, T. (2021). Therapeutic Antibodies for the Treatment of Respiratory Tract Infections-Current Overview and Perspectives. Vaccines *9*, 151. https://doi.org/10.3390/vaccines9020151.
5. Nesbitt, H., Burke, C., and Haghi, M. (2021). Manipulation of the Upper Respiratory Microbiota to Reduce Incidence and Severity of Upper Respiratory Viral Infections: A Literature Review. Front. Microbiol. *12*, 713703. https://doi.org/10.3389/fmicb.2021.713703.
6. Nutrition Division (2006). Probiotics in Food: Health and Nutritional Properties and Guidelines for Evaluation - Report of a Joint FAO/WHO Expert Consultation on Evaluation of Health and Nutritional Properties of Probiotics in Food Including Powder Milk with Live Lactic Acid Bacteria (FAO/WHO).
7. Manzanares, W., Lemieux, M., Langlois, P.L., and Wischmeyer, P.E. (2016). Probiotic and synbiotic therapy in critical illness: a systematic review and meta-analysis. Crit. Care *19*, 262. https://doi.org/10.1186/s13054-016-1434-y.
8. Batra, P., Soni, K.D., and Mathur, P. (2020). Efficacy of probiotics in the prevention of VAP in critically ill ICU patients: an updated systematic review and meta-analysis of randomized control trials. J. Intensive Care *8*, 81. https://doi.org/10.1186/s40560-020-00487-8.
9. Roquilly, A., Torres, A., Villadangos, J.A., Netea, M.G., Dickson, R., Becher, B., and Asehnoune, K. (2019). Pathophysiological role of respiratory dysbiosis in hospital-acquired pneumonia. Lancet Respir. Med. *7*, 710–720. https://doi.org/10.1016/S2213-2600(19)30140-7.
10. Zhao, Y., Dong, B.R., and Hao, Q. (2022). Probiotics for preventing acute upper respiratory tract infections. Cochrane Database Syst. Rev. *8*, CD006895. https://doi.org/10.1002/14651858.CD006895.pub4.
11. Long, J.D., and Morris, A. (2017). Probiotics in Preventing Acute Upper Respiratory Tract Infections. Am. J. Nurs. *117*, 69. https://doi.org/10.1097/01.NAJ.0000527494.16987.1e.
12. Laurenzi, G.A., Potter, R.T., and Kass, E.H. (1961). Bacteriologic flora of the lower respiratory tract. N. Engl. J. Med. *265*, 1273–1278. https://doi.org/10.1056/NEJM196112282652601.
13. Bernard-Raichon, L., Colom, A., Monard, S.C., Namouchi, A., Cescato, M., Garnier, H., Leon-Icaza, S.A., Métais, A., Dumas, A., Corral, D., et al. (2021). A Pulmonary Lactobacillus murinus Strain Induces Th17 and RORgammat(+) Regulatory T Cells and Reduces Lung Inflammation in Tuberculosis. J. Immunol. *207*, 1857–1870. https://doi.org/10.4049/jimmunol.2001044.
14. Mathieu, E., MacPherson, C.W., Belvis, J., Mathieu, O., Robert, V., Saint-Criq, V., Langella, P., Tompkins, T.A., and Thomas, M. (2020). Oral Primo-Colonizing Bacteria Modulate Inflammation and Gene Expression in Bronchial Epithelial Cells. Microorganisms *8*, 1094. https://doi.org/10.3390/microorganisms8081094.
15. Branch-Elliman, W., Wright, S.B., and Howell, M.D. (2015). Determining the Ideal Strategy for Ventilator-associated Pneumonia Prevention. Cost-Benefit Analysis. Am. J. Respir. Crit. Care Med. *192*, 57–63. https://doi.org/10.1164/rccm.201412-2316OC.
16. McClave, S.A., Taylor, B.E., Martindale, R.G., Warren, M.M., Johnson, D.R., Braunschweig, C., McCarthy, M.S., Davanos, E., Rice, T.W., Cresci, G.A., et al. (2016). Guidelines for the Provision and Assessment of Nutrition Support Therapy in the Adult Critically Ill Patient: Society of Critical Care Medicine (SCCM) and American Society for Parenteral and Enteral Nutrition (A.S.P.E.N.). JPEN - J. Parenter. Enter. Nutr. *40*, 159–211. https://doi.org/10.1177/0148607115621863.
17. Johnstone, J., Meade, M., Lauzier, F., Marshall, J., Duan, E., Dionne, J., Arabi, Y.M., Heels-Ansdell, D., Thabane, L., Lamarche, D., et al. (2021). Effect of Probiotics on Incident Ventilator-Associated Pneumonia in Critically Ill Patients: A Randomized Clinical Trial. JAMA *326*, 1024–1033. https://doi.org/10.1001/jama.2021.13355.

18. Maillet, A., Guilleminault, L., Lemarié, E., Lerondel, S., Azzopardi, N., Montharu, J., Congy-Jolivet, N., Reverdiau, P., Legrain, B., Parent, C., et al. (2011). The airways, a novel route for delivering monoclonal antibodies to treat lung tumors. Pharm. Res. (N. Y.) *28*, 2147–2156. https://doi.org/10.1007/s11095-011-0442-5.

19. Respaud, R., Vecellio, L., Diot, P., and Heuzé-Vourc'h, N. (2015). Nebulization as a delivery method for mAbs in respiratory diseases. Expet Opin. Drug Deliv. *12*, 1027–1039. https://doi.org/10.1517/17425247.2015.999039.

20. Respaud, R., Marchand, D., Pelat, T., Tchou-Wong, K.M., Roy, C.J., Parent, C., Cabrera, M., Guillemain, J., Mac Loughlin, R., Levacher, E., et al. (2016). Development of a drug delivery system for efficient alveolar delivery of a neutralizing monoclonal antibody to treat pulmonary intoxication to ricin. J. Contr. Release *234*, 21–32. https://doi.org/10.1016/j.jconrel.2016.05.018.

21. Guilleminault, L., Azzopardi, N., Arnoult, C., Sobilo, J., Hervé, V., Montharu, J., Guillon, A., Andres, C., Herault, O., Le Pape, A., et al. (2014). Fate of inhaled monoclonal antibodies after the deposition of aerosolized particles in the respiratory system. J. Contr. Release *196*, 344–354. https://doi.org/10.1016/j.jconrel.2014.10.003.

22. Sécher, T., Dalonneau, E., Ferreira, M., Parent, C., Azzopardi, N., Paintaud, G., Si-Tahar, M., and Heuzé-Vourc'h, N. (2019). In a murine model of acute lung infection, airway administration of a therapeutic antibody confers greater protection than parenteral administration. J. Contr. Release *303*, 24–33. https://doi.org/10.1016/j.jconrel.2019.04.005.

23. Frank, D.W., Vallis, A., Wiener-Kronish, J.P., Roy-Burman, A., Spack, E.G., Mullaney, B.P., Megdoud, M., Marks, J.D., Fritz, R., and Sawa, T. (2002). Generation and characterization of a protective monoclonal antibody to Pseudomonas aeruginosa PcrV. J. Infect. Dis. *186*, 64–73. https://doi.org/10.1086/341069.

24. Faure, K., Fujimoto, J., Shimabukuro, D.W., Ajayi, T., Shime, N., Moriyama, K., Spack, E.G., Wiener-Kronish, J.P., and Sawa, T. (2003). Effects of monoclonal anti-PcrV antibody on Pseudomonas aeruginosa-induced acute lung injury in a rat model. J. Immune Base Ther. Vaccine *1*, 2. https://doi.org/10.1186/1476-8518-1-2.

25. Leyva-Grado, V.H., Tan, G.S., Leon, P.E., Yondola, M., and Palese, P. (2015). Direct administration in the respiratory tract improves efficacy of broadly neutralizing anti-influenza virus monoclonal antibodies. Antimicrob. Agents Chemother. *59*, 4162–4172. https://doi.org/10.1128/AAC.00290-15.

26. Piepenbrink, M.S., Park, J.G., Oladunni, F.S., Deshpande, A., Basu, M., Sarkar, S., Loos, A., Woo, J., Lovalenti, P., Sloan, D., et al. (2021). Therapeutic activity of an inhaled potent SARS-CoV-2 neutralizing human monoclonal antibody in hamsters. Cell Rep. Med. *2*, 100218. https://doi.org/10.1016/j.xcrm.2021.100218.

27. Secher, T., Dalonneau, E., Ferreira, M., Parent, C., Azzopardi, N., Paintaud, G., Si-Tahar, M., and Heuze-Vourc'h, N. (2019). In a murine model of acute lung infection, airway administration of a therapeutic antibody confers greater protection than parenteral administration. J. Contr. Release *303*, 24–33. https://doi.org/10.1016/j.jconrel.2019.04.005.

28. Vigil, A., Frias-Staheli, N., Carabeo, T., and Wittekind, M. (2020). Airway Delivery of Anti-influenza Monoclonal Antibodies Results in Enhanced Antiviral Activities and Enables Broad-Coverage Combination Therapies. J. Virol. *94*, e00052-20. https://doi.org/10.1128/JVI.00052-20.

29. Remot, A., Descamps, D., Noordine, M.L., Boukadiri, A., Mathieu, E., Robert, V., Riffault, S., Lambrecht, B., Langella, P., Hammad, H., and Thomas, M. (2017). Bacteria isolated from lung modulate asthma susceptibility in mice. ISME J. *11*, 1061–1074. https://doi.org/10.1038/ismej.2016.181.

30. Klastersky, J., and Zinner, S.H. (1982). Synergistic combinations of antibiotics in gram-negative bacillary infections. Rev. Infect. Dis. *4*, 294–301. https://doi.org/10.1093/clinids/4.2.294.

31. Secher, T., Fas, S., Fauconnier, L., Mathieu, M., Rutschi, O., Ryffel, B., and Rudolf, M. (2013). The Anti-Pseudomonas aeruginosa Antibody Panobacumab Is Efficacious on Acute Pneumonia in Neutropenic Mice and Has Additive Effects with Meropenem. PLoS One *8*, e73396.

32. Cripps, A.W., Dunkley, M.L., Clancy, R.L., and Kyd, J. (1995). Pulmonary immunity to Pseudomonas aeruginosa. Immunol Cell Biol. *73*, 418–424. https://doi.org/10.1038/icb.1995.65.

33. Lavoie, E.G., Wangdi, T., and Kazmierczak, B.I. (2011). Innate immune responses to Pseudomonas aeruginosa infection. Microb. Infect. *13*, 1133–1145. https://doi.org/10.1016/j.micinf.2011.07.011.

34. Lin, C.K., and Kazmierczak, B.I. (2017). Inflammation: A Double-Edged Sword in the Response to Pseudomonas aeruginosa Infection. J. Innate Immun. *9*, 250–261. https://doi.org/10.1159/000455857.

35. Pitiot, A., Ferreira, M., Parent, C., Boisseau, C., Cortes, M., Bouvart, L., Paget, C., Heuzé-Vourc'h, N., and Sécher, T. (2023). Mucosal administration of anti-bacterial antibodies provide long-term cross-protection against Pseudomonas aeruginosa respiratory infection. Mucosal Immunol. *16*, 312–325. https://doi.org/10.1016/j.mucimm.2023.03.005.

36. Ye, J., Shao, H., Hickman, D., Angel, M., Xu, K., Cai, Y., Song, H., Fouchier, R.A.M., Qin, A., and Perez, D.R. (2010). Intranasal delivery of an IgA monoclonal antibody effective against sublethal H5N1 influenza virus infection in mice. Clin. Vaccine Immunol. *17*, 1363–1370. https://doi.org/10.1128/CVI.00002-10.

37. Proctor, L.L., Ward, W.L., Roggy, C.S., Koontz, A.G., Clark, K.M., Quinn, A.P., Schroeder, M., Brooks, A.E., Small, J.M., Towne, F.D., and Brooks, B.D. (2021). Potential Therapeutic Targets for Combination Antibody Therapy against Pseudomonas aeruginosa Infections. Antibiotics *10*, 1530. https://doi.org/10.3390/antibiotics10121530.

38. Domenech, M., Sempere, J., de Miguel, S., and Yuste, J. (2018). Combination of Antibodies and Antibiotics as a Promising Strategy Against Multidrug-Resistant Pathogens of the Respiratory Tract. Front. Immunol. *9*, 2700. https://doi.org/10.3389/fimmu.2018.02700.

39. Laiño, J., Villena, J., Suvorov, A., Zelaya, H., Ortiz Moyano, R., Salva, S., and Alvarez, S. (2018). Nasal immunization with recombinant chimeric pneumococcal protein and cell wall from immunobiotic bacteria improve resistance of infant mice to Streptococcus pneumoniae infection. PLoS One *13*, e0206661. https://doi.org/10.1371/journal.pone.0206661.

40. Zhu, Y., Tao, X., Yan, T., Cao, S., Jiang, P., Zhang, Z., Li, L., and Wu, Q. (2024). Lactobacillus murinus alleviated lung inflammation induced by PAHs in mice. Ecotoxicol. Environ. Saf. *281*, 116662. https://doi.org/10.1016/j.ecoenv.2024.116662.

41. Yeo, J.-M., Lee, H.-J., Kim, J.-W., Lee, J.-B., Park, S.-Y., Choi, I.-S., and Song, C.-S. (2014). Lactobacillus fermentum CJL-112 protects mice against influenza virus infection by activating T-helper 1 and eliciting a protective immune response. Int. Immunopharm. *18*, 50–54. https://doi.org/10.1016/j.intimp.2013.10.020.

42. Youn, H.N., Lee, D.H., Lee, Y.N., Park, J.K., Yuk, S.S., Yang, S.Y., Lee, H.J., Woo, S.H., Kim, H.M., Lee, J.B., et al. (2012). Intranasal administration of live Lactobacillus species facilitates protection against influenza virus infection in mice. Antivir. Res. *93*, 138–143. https://doi.org/10.1016/j.antiviral.2011.11.004.

43. Dyer, K.D., Drummond, R.A., Rice, T.A., Percopo, C.M., Brenner, T.A., Barisas, D.A.G., Karpe, K.A., Moore, M.L., and Rosenberg, H.F. (2016). Priming of the Respiratory Tract with Immunobiotic Lactobacillus plantarum Limits Infection of Alveolar Macrophages with Recombinant Pneumonia Virus of Mice (rK2-PVM). J. Virol. *90*, 979–991. https://doi.org/10.1128/JVI.02279-15.

44. Cangemi de Gutierrez, R.C., Santos de Araoz, V.S., and Nader-Macías, M.E. (2000). Effect of intranasal administration of Lactobacillus fermentum on the respiratory tract of mice. Biol. Pharm. Bull. *23*, 973–978. https://doi.org/10.1248/bpb.23.973.

45. Doron, S., and Snydman, D.R. (2015). Risk and safety of probiotics. Clin. Infect. Dis. *60*, S129–S134. https://doi.org/10.1093/cid/civ085.

46. Hempel, S., Newberry, S., Ruelaz, A., Wang, Z., Miles, J.N.V., Suttorp, M.J., Johnsen, B., Shanman, R., Slusser, W., Fu, N., et al. (2011). Safety of probiotics used to reduce risk and prevent or treat disease. Evid. Rep. Technol. Assess. *200*, 1–645.

47. Langella, P., and Chatel, J.M. (2019). Risk assessment of probiotics use requires clinical parameters. Nat. Rev. Gastroenterol. Hepatol. *16*, 202–204. https://doi.org/10.1038/s41575-019-0111-4.

48. Rosas-Salazar, C., Shilts, M.H., Tovchigrechko, A., Schobel, S., Chappell, J.D., Larkin, E.K., Gebretsadik, T., Halpin, R.A., Nelson, K.E., Moore, M.L., et al. (2018). Nasopharyngeal Lactobacillus is associated with a reduced risk of childhood wheezing illnesses following acute respiratory syncytial virus infection in infancy. J. Allergy Clin. Immunol. *142*, 1447–1456.e9. https://doi.org/10.1016/j.jaci.2017.10.049.

49. Salminen, M.K., Rautelin, H., Tynkkynen, S., Poussa, T., Saxelin, M., Valtonen, V., and Järvinen, A. (2004). Lactobacillus bacteremia, clinical significance, and patient outcome, with special focus on probiotic L. rhamnosus GG. Clin. Infect. Dis. *38*, 62–69. https://doi.org/10.1086/380455.

50. Chaini, E., Chainis, N.D., Ioannidis, A., Magana, M., Nikolaou, C., Papaparaskevas, J., Liakata, M.V., Katopodis, P., Papastavrou, L., Tegos, G.P., and Chatzipanagiotou, S. (2016). Pneumonia and Pleural Empyema due to a Mixed Lactobacillus spp. Infection as a Possible Early Esophageal Carcinoma Signature. Front. Med. *3*, 42. https://doi.org/10.3389/fmed.2016.00042.

51. Forsythe, P. (2014). Probiotics and lung immune responses. Ann. Am. Thorac. Soc. *11*, S33–S37. https://doi.org/10.1513/AnnalsATS.201306-156MG.

52. Mizgerd, J.P. (2002). Molecular mechanisms of neutrophil recruitment elicited by bacteria in the lungs. Semin. Immunol. *14*, 123–132. https://doi.org/10.1006/smim.2001.0349.

53. Goodman, R.B., Strieter, R.M., Martin, D.P., Steinberg, K.P., Milberg, J.A., Maunder, R.J., Kunkel, S.L., Walz, A., Hudson, L.D., and Martin, T.R. (1996). Inflammatory cytokines in patients with persistence of the acute respiratory distress syndrome. Am. J. Respir. Crit. Care Med. *154*, 602–611. https://doi.org/10.1164/ajrccm.154.3.8810593.

54. Lebeer, S., Claes, I., Tytgat, H.L.P., Verhoeven, T.L.A., Marien, E., von Ossowski, I., Reunanen, J., Palva, A., Vos, W.M.d., Keersmaecker, S.C.J.D., and Vanderleyden, J. (2012). Functional analysis of Lactobacillus rhamnosus GG pili in relation to adhesion and immunomodulatory interactions with intestinal epithelial cells. Appl. Environ. Microbiol. *78*, 185–193. https://doi.org/10.1128/AEM.06192-11.

55. Plaza-Diaz, J., Ruiz-Ojeda, F.J., Gil-Campos, M., and Gil, A. (2019). Mechanisms of Action of Probiotics. Adv. Nutr. *10*, S49–S66. https://doi.org/10.1093/advances/nmy063.

56. York, A. (2018). Microbiome: Gut microbiota sways response to cancer immunotherapy. Nat. Rev. Microbiol. *16*, 121. https://doi.org/10.1038/nrmicro.2018.12.

57. West, N.R., and Powrie, F. (2015). Immunotherapy Not Working? Check Your Microbiota. Cancer Cell *28*, 687–689. https://doi.org/10.1016/j.ccell.2015.11.010.

58. Kumova, O.K., Fike, A.J., Thayer, J.L., Nguyen, L.T., Mell, J.C., Pascasio, J., Stairiker, C., Leon, L.G., Katsikis, P.D., and Carey, A.J. (2019). Lung transcriptional unresponsiveness and loss of early influenza virus control in infected neonates is prevented by intranasal Lactobacillus rhamnosus GG. PLoS Pathog. *15*, e1008072. https://doi.org/10.1371/journal.ppat.1008072.

59. Fangous, M.S., Gosset, P., Galakhoff, N., Gouriou, S., Guilloux, C.A., Payan, C., Vallet, S., Héry-Arnaud, G., and Le Berre, R. (2021). Priming with intranasal lactobacilli prevents Pseudomonas aeruginosa acute pneumonia in mice. BMC Microbiol. *21*, 195. https://doi.org/10.1186/s12866-021-02254-7.

60. Vetizou, M., Pitt, J.M., Daillere, R., Lepage, P., Waldschmitt, N., Flament, C., Rusakiewicz, S., Routy, B., Roberti, M.P., Duong, C.P., et al. (2015). Anticancer immunotherapy by CTLA-4 blockade relies on the gut microbiota. Science *350*, 1079–1084. https://doi.org/10.1126/science.aad1329.

61. Zitvogel, L., Ma, Y., Raoult, D., Kroemer, G., and Gajewski, T.F. (2018). The microbiome in cancer immunotherapy: Diagnostic tools and therapeutic strategies. Science *359*, 1366–1370. https://doi.org/10.1126/science.aar6918.

62. Baruch, E.N., Wang, J., and Wargo, J.A. (2021). Gut Microbiota and Antitumor Immunity: Potential Mechanisms for Clinical Effect. Cancer Immunol. Res. *9*, 365–370. https://doi.org/10.1158/2326-6066.CIR-20-0877.

63. Tonetti, F.R., Islam, M.A., Vizoso-Pinto, M.G., Takahashi, H., Kitazawa, H., and Villena, J. (2020). Nasal priming with immunobiotic lactobacilli improves the adaptive immune response against influenza virus. Int. Immunopharm. *78*, 106115. https://doi.org/10.1016/j.intimp.2019.106115.

64. Friedland, I., Gallagher, G., King, T., and Woods, G.L. (2004). Antimicrobial susceptibility patterns in Pseudomonas aeruginosa: data from a multicenter Intensive Care Unit Surveillance Study (ISS) in the United States. J. Chemother. *16*, 437–441. https://doi.org/10.1179/joc.2004.16.5.437.

65. Chastre, J., and Fagon, J.Y. (2002). Ventilator-associated pneumonia. Am. J. Respir. Crit. Care Med. *165*, 867–903. https://doi.org/10.1164/ajrccm.165.7.2105078.

66. El Solh, A.A., and Alhajhusain, A. (2009). Update on the treatment of Pseudomonas aeruginosa pneumonia. J. Antimicrob. Chemother. *64*, 229–238. https://doi.org/10.1093/jac/dkp201.

67. Gadre, S.K., Shah, M., Mireles-Cabodevila, E., Patel, B., and Duggal, A. (2019). Epidemiology and Predictors of 30-Day Readmission in Patients With Sepsis. Chest *155*, 483–490. https://doi.org/10.1016/j.chest.2018.12.008.

68. Zheng, J., Wittouck, S., Salvetti, E., Franz, C.M.A.P., Harris, H.M.B., Mattarelli, P., O'Toole, P.W., Pot, B., Vandamme, P., Walter, J., et al. (2020). A taxonomic note on the genus Lactobacillus: Description of 23 novel genera, emended description of the genus Lactobacillus Beijerinck 1901, and union of Lactobacillaceae and Leuconostocaceae. Int. J. Syst. Evol. Microbiol. *70*, 2782–2858. https://doi.org/10.1099/ijsem.0.004107.

69. Reygner, J., Charrueau, C., Delannoy, J., Mayeur, C., Robert, V., Cuinat, C., Meylheuc, T., Mauras, A., Augustin, J., Nicolis, I., et al. (2020). Freeze-dried fecal samples are biologically active after long-lasting storage and suited to fecal microbiota transplantation in a preclinical murine model of Clostridioides difficile infection. Gut Microb. *11*, 1405–1422. https://doi.org/10.1080/19490976.2020.1759489.

70. Salandre, A., Delannoy, J., Goudiaby, M.T.B., Barbut, F., Thomas, M., Waligora-Dupriet, A.-J., and Kapel, N. (2023). A Simple In Vitro Test to Select Stools for Fecal Microbiota Transplantation to Limit Intestinal Carriage of Extensively Drug-Resistant Bacteria. Microorganisms *11*, 2753. https://doi.org/10.3390/microorganisms11112753.

71. Deriu, E., Liu, J.Z., Pezeshki, M., Edwards, R.A., Ochoa, R.J., Contreras, H., Libby, S.J., Fang, F.C., and Raffatellu, M. (2013). Probiotic bacteria reduce salmonella typhimurium intestinal colonization by competing for iron. Cell Host Microbe *14*, 26–37. https://doi.org/10.1016/j.chom.2013.06.007.

72. Massip, C., Branchu, P., Bossuet-Greif, N., Chagneau, C.V., Gaillard, D., Martin, P., Boury, M., Sécher, T., Dubois, D., Nougayrède, J.-P., and Oswald, E. (2019). Deciphering the interplay between the genotoxic and probiotic activities of Escherichia coli Nissle 1917. PLoS Pathog. *15*, e1008029. https://doi.org/10.1371/journal.ppat.1008029.

73. Valdéz, J.C., Peral, M.C., Rachid, M., Santana, M., and Perdigón, G. (2005). Interference of Lactobacillus plantarum with Pseudomonas aeruginosa in vitro and in infected burns: the potential use of probiotics in wound treatment. Clin. Microbiol. Infect. *11*, 472–479. https://doi.org/10.1111/j.1469-0691.2005.01142.x.

OMTM, Volume *32*

Supplemental information

Synergy between *Lactobacillus murinus* and anti-PcrV antibody delivered in the airways to boost protection against *Pseudomonas aeruginosa*

Thomas Sécher, Mélanie Cortes, Chloé Boisseau, Marie-Thérèse Barba Goudiaby, Aubin Pitiot, Christelle Parent, Muriel Thomas, and Nathalie Heuzé-Vourc'h

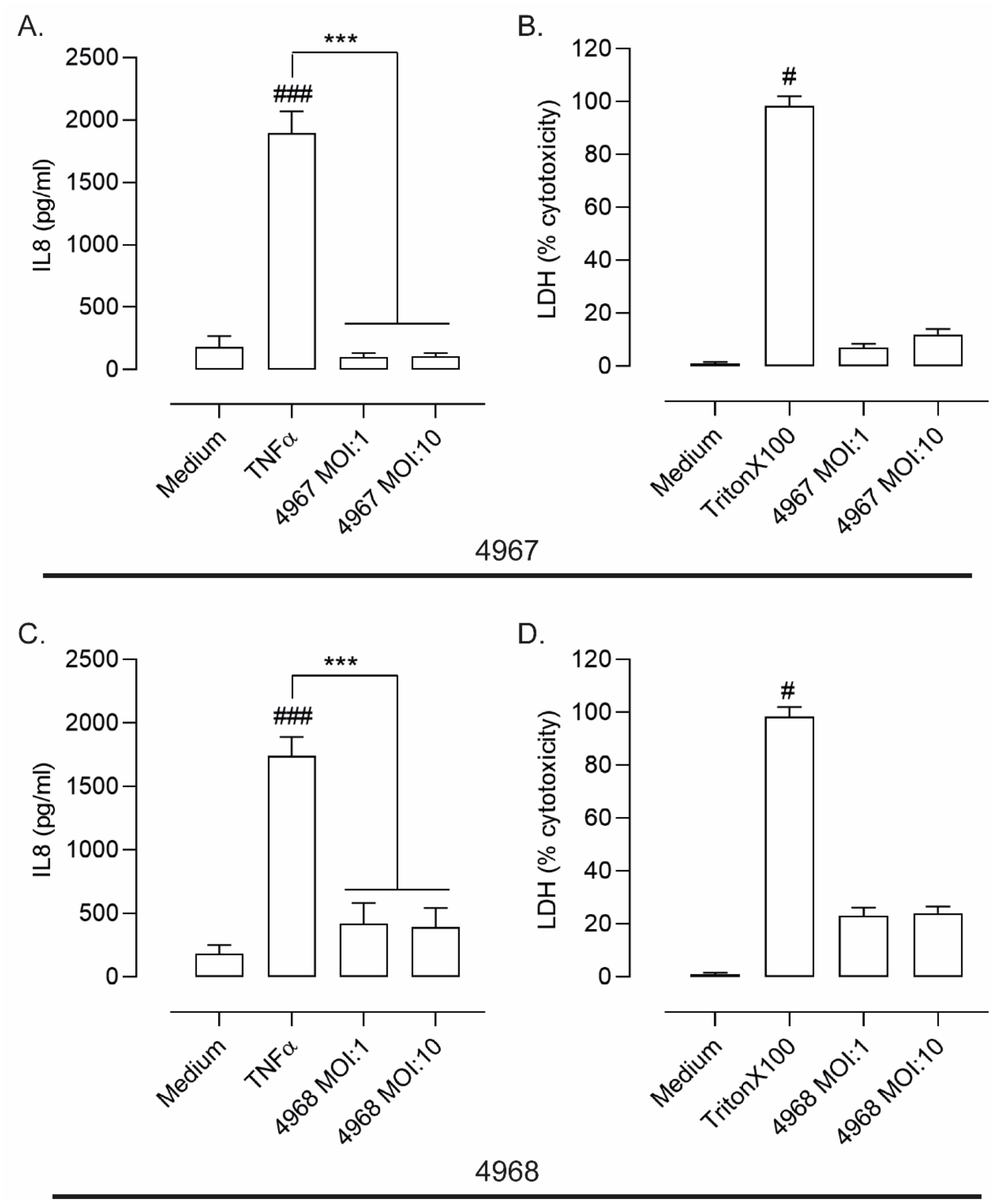

Figure S1: BEAS-2B cells were exposed or not with CNCM-I 4967 (MOI:1 or MOI:10), CNCM-I 4968 (MOI:1 or MOI:10), TNF (1ng/mL) or TritonX100 (10%) for 18h. Production of IL-8 (A and C) and of LDH (B and D) were measured. LDH was expressed as % of response over Triton-X100 conditions (positive control for cell death. The data are quoted as the mean values ± SEM. The results correspond to 3 pooled, independent experiments (n=3-9 per conditions), #: p< 0.05, ###: p<0.001 when compared to medium condition; ***: p<0.001 when compared TritonX100 condition.

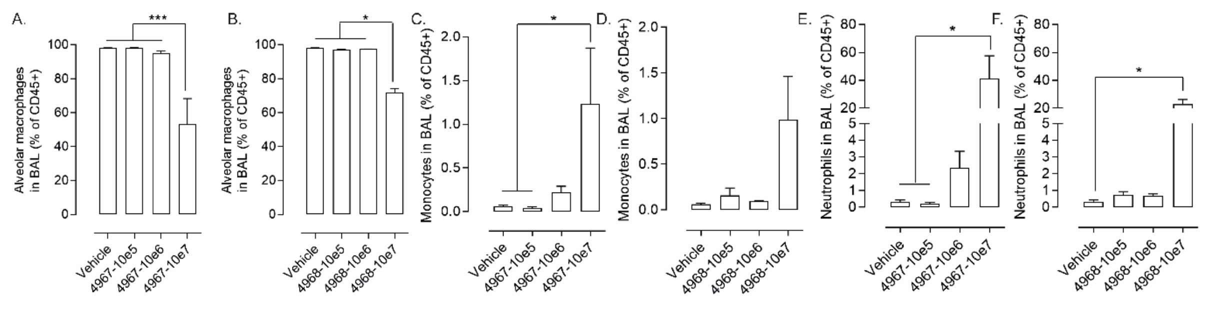

<u>Figure S2:</u> Mice were treated as described in Figure 2A. One day later, mice were sacrificed. Frequency of alveolar macrophages (CD45+ CD11c+ CD11b- SiglecF+ Ly6G- Ly6C- cells) (A and B), monocytes (CD45+ CD11c- CD11b+ SiglecF+ Ly6G- Ly6C+) (C and D) and neutrophils (CD45+ CD11c- CD11b+ SiglecF- Ly6G+ Ly6C-) (E and F) were determined in BAL. The data are quoted as the mean values ± SEM. The results are representative of 2 independent experiments (n=4-5 mice per group), *: $p<0.05$, ***: $p<0.001$ with *t* test.

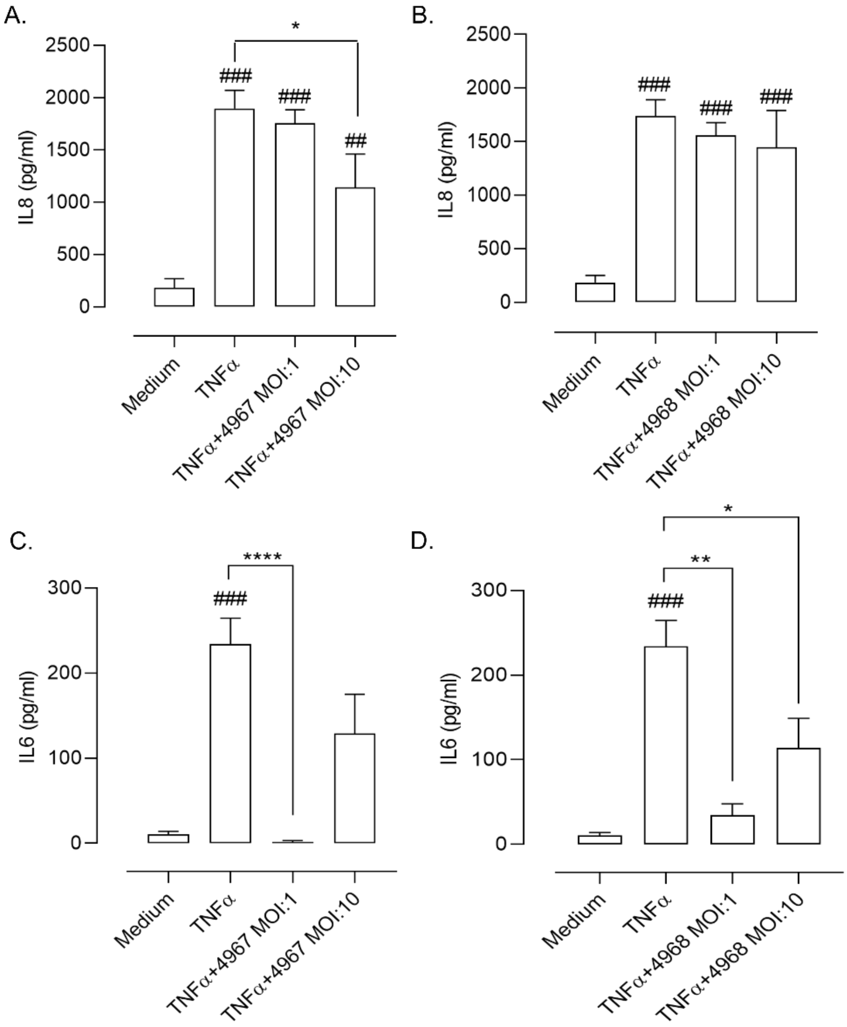

Figure S3: BEAS-2B cells were exposed or not with CNCM-I 4967 (MOI:1 or MOI:10), CNCM-I 4968 (MOI:1 or MOI:10) and/or TNF (1ng/mL) for 18h. Production of IL-8 (A and B) and IL-6 (C and D) was measured. The data are quoted as the mean values ± SEM. The results correspond to 3 pooled, independent experiments (n=3-9 per conditions), ##: $p< 0.01$, ###: $p<0.001$ when compared to medium condition; ***: $p<0.001$ when compared TNF condition.

A.

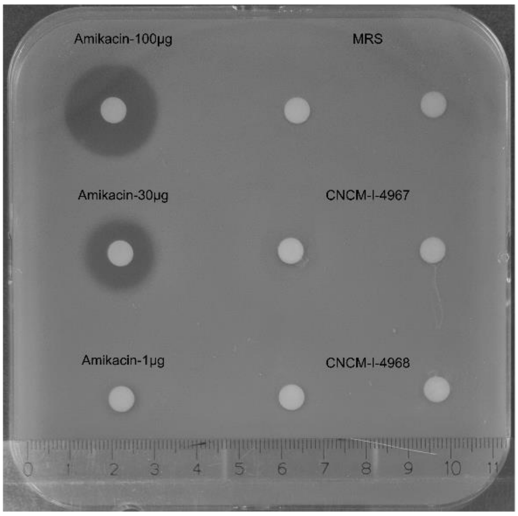

B.

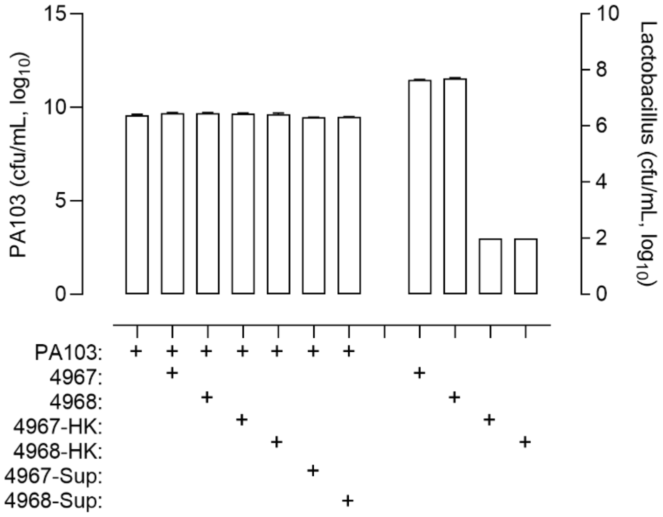

<u>Figure S4:</u> (A) Picture of growth inhibition zone induced by CNCM-I 4967, CNCM-I 4968 or Amikacin (1, 30, 100μg) on *P. aeruginosa* PA103 cultured in soft-agar. (B) CFU counts of *P. aeruginosa* PA103 following a 24-hour co-culture in LB medium with live CNCM-I 4967 (4967), live CNCM-I 4968 (4968), heat-killed CNCM-I 4967 (4967-HK), heat-killed CNCM-I 4968 (4968-HK), supernatant from live CNCM-I 4967 (4967-Sup), supernatant from live CNCM-I 4968 (4968-Sup) have been evaluated on PIA-agar and depicted on the left-side axis. CFU counts of CNCM-I 4967 (4967), live CNCM-I 4968 (4968), heat-killed CNCM-I 4967 (4967-HK), heat-killed CNCM-I 4968 (4968-HK) have been evaluated on MRS-agar and depicted on the right-side axis. The data are quoted as the mean values ± SEM. The results are representative of two independent experiments.

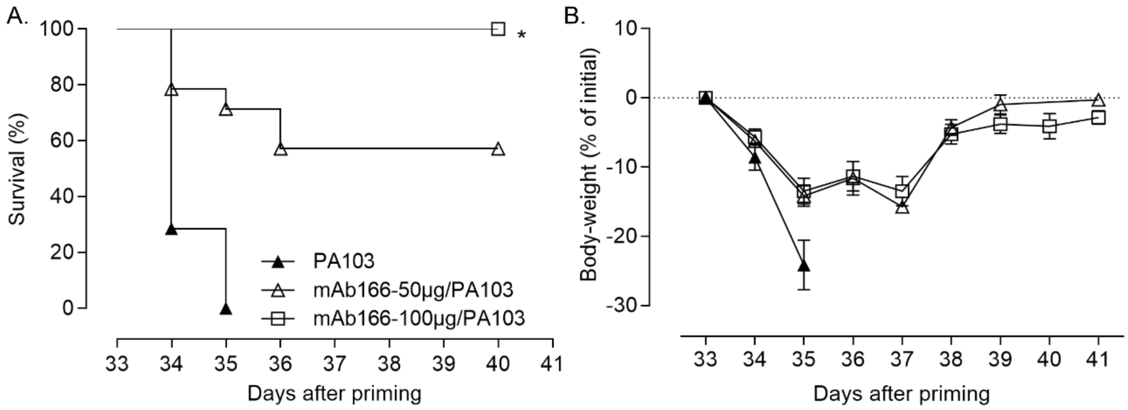

Figure S5: Mice were treated as described in Figure 5A. Survival (A) and body-weight (B) were monitored after the secondary infection. The results correspond to 4 pooled, independent experiments (n=7-20 mice per group). Log-rank test was used for survival analysis, *: p<0.05.
